# Differential Network Testing Reveals Diverging Dynamics of Organ System Interactions for Survivors and Non-survivors in Intensive Care Medicine

**DOI:** 10.3389/fphys.2021.801622

**Published:** 2022-01-10

**Authors:** Roman Schefzik, Leonie Boland, Bianka Hahn, Thomas Kirschning, Holger A. Lindner, Manfred Thiel, Verena Schneider-Lindner

**Affiliations:** ^1^Department of Anesthesiology and Surgical Intensive Care Medicine, Medical Faculty Mannheim, Heidelberg University, Mannheim, Germany; ^2^Mannheim Institute of Innate Immunoscience (MI3), Medical Faculty Mannheim, Heidelberg University, Mannheim, Germany; ^3^Department of Community Health Sciences, Max Rady College of Medicine, University of Manitoba, Winnipeg, MB, Canada

**Keywords:** correlation, cross-sectional study, intensive care medicine, longitudinal study, network comparison, permutation test

## Abstract

Statistical network analyses have become popular in many scientific disciplines, where an important task is to test for differences between two networks. We describe an overall framework for differential network testing procedures that vary regarding (1) the network estimation method, typically based on specific concepts of association, and (2) the network characteristic employed to measure the difference. Using permutation-based tests, our approach is general and applicable to various overall, node-specific or edge-specific network difference characteristics. The methods are implemented in our freely available R software package DNT, along with an R Shiny application. In a study in intensive care medicine, we compare networks based on parameters representing main organ systems to evaluate the prognosis of critically ill patients in the intensive care unit (ICU), using data from the surgical ICU of the University Medical Centre Mannheim, Germany. We specifically consider both cross-sectional comparisons between a non-survivor and a survivor group and longitudinal comparisons at two clinically relevant time points during the ICU stay: first, at admission, and second, at an event stage prior to death in non-survivors or a matching time point in survivors. The non-survivor and the survivor networks do not significantly differ at the admission stage. However, the organ system interactions of the survivors then stabilize at the event stage, revealing significantly more network edges, whereas those of the non-survivors do not. In particular, the liver appears to play a central role for the observed increased connectivity in the survivor network at the event stage.

## Introduction

**Figure 1:**
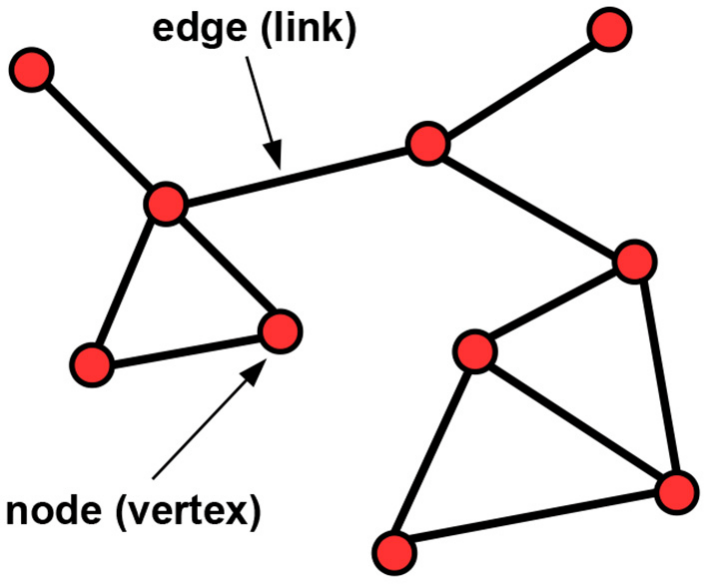
Illustration of a network.

Statistical network analyses (Newman, 2018) have become popular in many scientific disciplines, such as life science (Mathews et al., 2020; Sinkala et al., 2020), social sciences (Baggio et al., 2016), psychology (Epskamp et al., 2018a) or political sciences (Porter et al., 2005). A network or graph (Kolaczyk, 2009; Hevey, 2018; Newman, 2018) is typically specified by a set of nodes (vertices) and edges (links), see Figure 1 for an illustration, and there are multiple possibilities to estimate it. An important task is to test for differences between two networks (Lichtblau et al., 2017), specified in terms of various network difference characteristics, which may refer to overall, node-specific or edge-specific differences. Testing for differences between two networks has previously been addressed, among others, in applications in psychology (Van Borkulo et al., 2017) and in the context of microbiome data (Peschel et al., 2021), biological interaction networks (Ali et al., 2014; Kuntal et al., 2016) or gene expression analysis (Gonzalez-Valbuena and Treviño, 2017).

The aim of our paper is twofold:

1. We first present a general framework for differential network testing, in which we review and fuse various options for (1) network estimation and (2) specifying a difference characteristic according to which a difference is measured. In particular, we check for significant differences between two networks using a generally applicable permutation test-based procedure (Good, 2013). The approaches are implemented in our R package DNT, referring to Differential Network Testing.
2. We then demonstrate the utility of differential network testing in a novel application to intensive care medicine, where network analyses do not have a long tradition.

**Figure 2:**
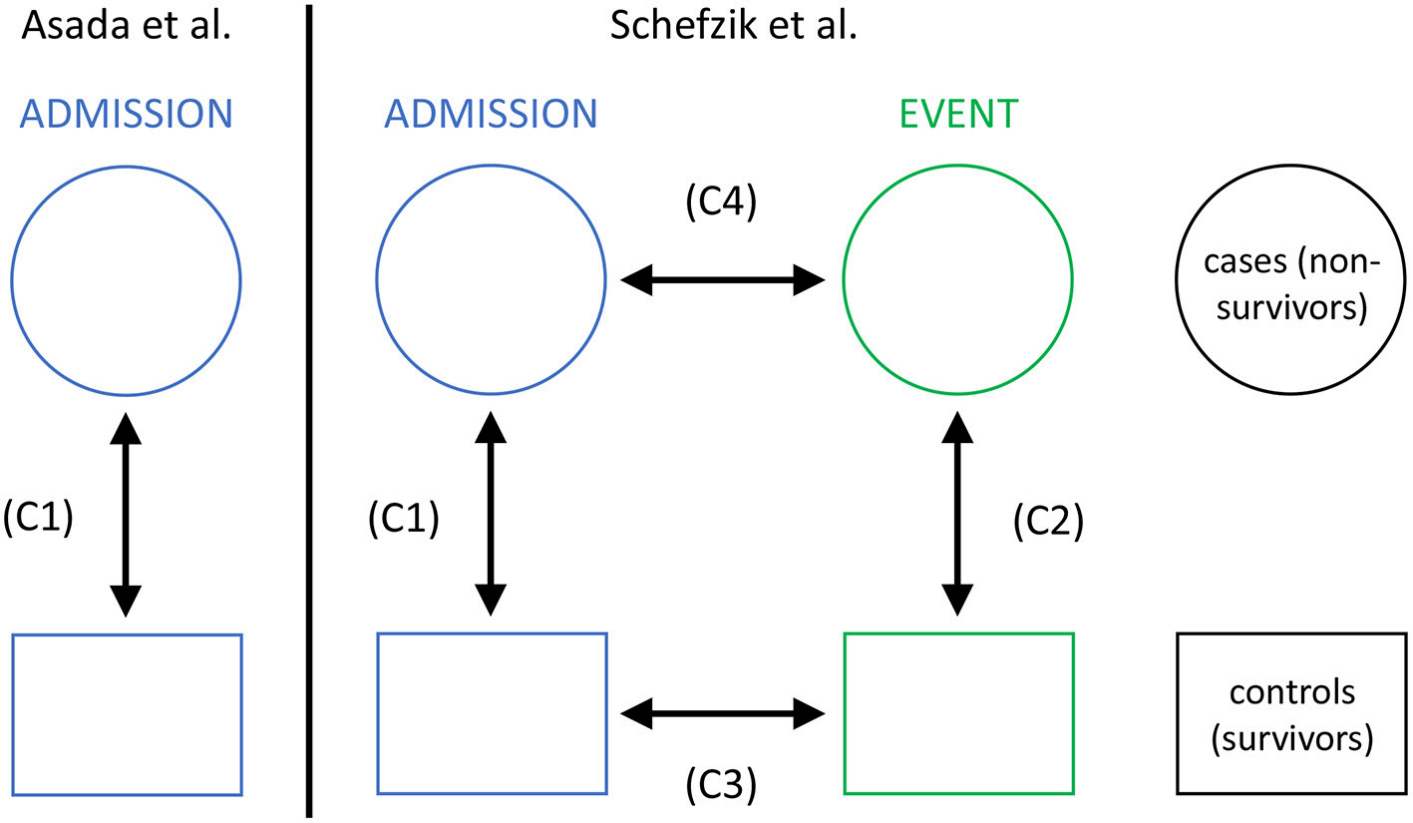
The setting of our study in intensive care medicine with cross-sectional (C1 and C2) and longitudinal (C3 and C4) comparisons, compared to that in Asada et al. (2016) with cross-sectional comparisons only (C1).

In this application, we specifically extend a previous study by Asada et al. (2016) and compare networks based on parameters representing main organ systems for a non-survivor and a matched survivor patient group from an intensive care unit (ICU). While Asada et al. performed a respective cross-sectional comparison at ICU admission stage only, in our observational study, we additionally consider a second clinically relevant stage, namely an event time point prior to death in non-survivors or a matching time point in survivors. Thus, we also perform longitudinal comparisons, allowing us to study the dynamics of organ system interactions in non-survivors and survivors in the course of ICU treatment (Figure 2).

## Materials and Methods

### Differential Network Testing

A network consisting of *N* nodes is typically summarized by a weighted adjacency matrix $A:={\left(a_{ij}\right)}_{i,j=1}^{N}$, where the edge weights *a*_*ij*_ between two nodes *i* and *j* refer to an associated value representing the magnitude or strength of an edge. When comparing two networks, a first step is to estimate each network (i.e., the edge weights) based on an (*S* × *N*) data matrix with *S* samples. To this end, different network estimation methods are available, and in what follows, we explicitly introduce those options of specifying the edge weights that are later used in our study.

- Edge weights can be given by the classical Spearman rank correlation coefficients with values in [−1, 1], covering negative and positive associations.
- Alternatively, the distance correlation (Székely et al., 2007; Edelmann et al., 2019, 2021) can be employed to define edge weights. This association measure, which takes values in [0, 1], can specify both linear and nonlinear dependence between two variables, and it is zero if and only if the two variables are independent.

In order to include only the most relevant edges into the network and avoid spurious ones, for the above approaches, an edge weight *a*_*ij*_ between two nodes *i* and *j* is set to zero, *a*_*ij*_: = 0, if the *p*-value referring to the corresponding association is larger than a specified threshold α (typically, α: = 0.05). Then, the association between *i* and *j* is not considered as significant and thus, no edge is drawn. In this context, *p*-values may be adjusted for multiple testing, e.g., via the established Bonferroni or Benjamini-Hochberg (BH) procedures.

Commonly, when estimating networks, there is a trade-off between sensitivity (drawing more edges at the cost of including spurious ones) and specificity (drawing fewer edges and thus avoiding spurious ones, at the cost of missing some "true" edges), and a prioritization may depend on the application at hand. In this context, drawing edges based on adjusted instead of unadjusted *p*-values can be expected to reduce the number of included edges. For this, the Bonferroni adjustment procedure is known as being more conservative than the BH adjustment procedure.

As an alternative to the above strategies, edge weights can be derived using the EBICglasso approach (Epskamp and Fried, 2018; Epskamp et al., 2018b; Hevey, 2018). This method combines network estimation based on partial correlations with lasso regularization (Tibshirani, 1996) and model selection. Here, partial correlation (Johnson and Wichern, 2002; Baba et al., 2004; Kim, 2015) quantifies the relationship between two variables while removing the effect of one or more additional variable(s) when assessing the correlation between the two variables and thus is an adjusted version of a classical (here, Spearman) correlation coefficient. In EBICglasso, lasso regularization is performed when estimating a network using partial correlations, where a collection of possible networks is estimated by varying the lasso tuning parameter λ. From this collection, a final “best” network is chosen by selecting a model based on the minimization of the extended Bayesian information criterion (EBIC) (Chen and Chen, 2008). In turn, the EBIC depends on a hyperparameter γ, typically γ ∈ [0, 0.5], which controls how much the EBIC prefers simpler models. Higher values of γ (~0.5) typically lead to a higher specificity in that more parsimonious models with fewer edges are preferred. In contrast, smaller values of γ (~ 0) typically lead to a higher sensitivity in that more edges are included into the network, thus being less conservative.

Once weighted adjacency matrices $A:={\left(a_{ij}\right)}_{i,j=1}^{N}$ and $B:={\left(b_{ij}\right)}_{i,j=1}^{N}$ representing two networks *A* and *B*, respectively, with the same nodes are determined, several network difference characteristics can be derived to compare *A* and *B* (Tantardini et al., 2019; Wills and Meyer, 2020; You, 2020). These may be roughly divided into differences in (1) overall network characteristics, (2) node-specific characteristics, or (3) edge-specific characteristics. From the large variety of network difference characteristics, we focus on the following well-established ten:

- difference in the global strength: $G\left(A,B\right):=|\overset{N}{\underset{i,j=1}{\sum }}\left(\left|a_{ij}|-|b_{ij}\right|\right)|$,
- Frobenius metric: $F\left(A,B\right):=\sqrt{\overset{N}{\underset{i,j=1}{\sum }}|a_{ij}-b_{ij}{|}^{2}}$,
- maximum metric: $M\left(A,B\right):=\underset{1\leq i,j\leq N}{\max}\left\{\left|a_{ij}-b_{ij}\right|\right\}$,
- spectral distance: $S\left(A,B\right):=\sqrt{\overset{N}{\underset{i=1}{\sum }}{\left(\lambda_{i}^{A}-\lambda_{i}^{B}\right)}^{2}}$, with $\lambda_{i}^{A}$ being the *i*-th eigenvalue of *A*, where $\lambda_{1}^{A}\geq \ldots \geq \lambda_{N}^{A}$,
- Jaccard distance:

$\begin{array}{c} J\left(A,B\right):=1-\frac{\#\text{edges that are present in A and B}}{\#\text{edges that are present in A or B}}\\ \;=1-\frac{\#\text{edges that are present in A and B}}{\#\text{edges}\left(A\right)+\#\text{edges}\left(B\right)-\#\text{edges that are present in A and B}} \end{array}$

- difference in the number of edges: *E*(*A, B*): = |#edges(*A*) − #edges(*B*)|,
- difference in the number of clusters (communities) obtained by the Girvan-Newman algorithm (Girvan and Newman, 2002; Newman and Girvan, 2004), which is based on the concept of edge betweenness (where the betweenness of an edge *e* refers to the number of the shortest paths between two nodes that go through *e*):
  *C*(*A, B*): = |#clusters(*A*) − #clusters(*B*)|,
- difference in the number of isolated nodes (i.e., nodes without any edges):
  *I*(*A, B*): = |#isolated nodes(*A*) − #isolated nodes(*B*)|,
- difference in the degree of a specific node *i*:
  *D*_*i*_(*A, B*): = |degree_*i*_(*A*) − degree_*i*_(*B*)|,
  where degree refers to the number of edges linked to *i*,
- difference in the edge strength between two specific nodes *i* and *j*: *E*_*ij*_(*A, B*): = |*a*_*ij*_ − *b*_*ij*_|.

While *G, F, M, S, J, E, C* and *I* can be considered as overall network difference characteristics, *D*_*i*_ is node-specific, and *E*_*ij*_ edge-specific. The above network difference characteristics represent quite basic measures, which are however well-established and readily interpretable and thus suitable to our application. In particular, *E, C, I, D*_*i*_ and *E*_*ij*_ are straightforward to interpret, directly representing differences of specific network properties. Further, *G* compares the respective sums of all (absolute) edge weights, and thus the global strengths, for two networks. Moreover, *F* and *M* can mathematically be regarded as overall distance measures induced by *p*-norms applied to adjacency matrices, using *p* = 2 (*F*) and *p* = ∞ (*M*), respectively. In addition, *S* is nothing but the Euclidean distance between spectra of adjacency matrices. Finally, *J*, which can take values in [0, 1], measures the dissimilarity between two networks by comparing the size of the intersection and the size of the union of their edges.

To test the null hypothesis *H*_0_ of invariance between two networks *A* and *B* with respect to a specific network difference characteristic (test statistic) *X* against the alternative *H*_1_ that there is a difference, we here use the following non-parametric, permutation-based procedure (Good, 2013; Van Borkulo et al., 2017):

1. based on the original group assignments of the samples to either network *A* or *B*, derive weighted adjacency matrices corresponding to *A* and *B*, respectively, and the value of the test statistic *x*_0_,
2. randomly permute the group assignments of the samples to either *A* or *B* and recalculate the weighted adjacency matrices and the value of the test statistic,
3. perform the preceding step *M* times, where *M* < *M*_all_ with *M*_all_ denoting the number of all possible permutations (as it is usually computationally infeasible to derive all possible permutations), and obtain the test statistic values *x*_1_, …, *x*_*M*_, and
4. derive an approximate *p*-value via $P=\left(1/M\right)\overset{M}{\underset{m=1}{\sum }}{𝟙}_{\left\{x_{m}\geq x_{0}\right\}}$, with 𝟙_*E*_ denoting the indicator function whose value is 1 if the event *E* materializes and 0 otherwise.

Then, the null hypothesis *H*_0_ can be rejected in favor of *H*_1_ if *P* ≤ α for some threshold α (typically, α = 0.05). The above strategy has the advantage of being quite general and generic, as it can be applied to any suitable network characteristic to compare two networks, including those mentioned above (*G, F, M, S, J, E, C, I, D*_*i*_ and *E*_*ij*_). Note that when permuting the group assignments, it needs be taken into account whether the data is paired (e.g., in longitudinal comparisons, where the measurements for both time points stem from the same individuals) or unpaired (e.g., in cross-sectional comparisons, where the measurements for both groups may come from different individuals). Typically, a large number of permutations is required to obtain reliable results, e.g., *M* = 1, 000 or *M* = 10, 000.

#### R Package DNT

The methodological framework introduced above is implemented in our new software package DNT in the R (R Core Team, 2021) programming language. DNT is publicly and freely available at https://github.com/RomanSchefzik/DNT, together with documentation and examples. In the context of statistical network comparison, it provides an alternative to the already existing R packages difconet (Gonzalez-Valbuena and Treviño, 2017), used for gene expression analysis, NCT (Van Borkulo et al., 2017), originally developed for applications in psychology, and NetCoMi (Peschel et al., 2021), specifically tailored to context of microbiome data. In accordance with what has been outlined above, DNT offers various options for specifying both the network estimation method and the network difference characteristics used for comparison. It is generally usable in any application area, whenever two statistical networks are to be compared. Along with the DNT package we additionally provide a user-friendly, interactive R Shiny application for visual network comparison as a special feature. This tool has the benefit of being readily employable by users with no (statistical) programming background.

### Application to Intensive Care Medicine

Organ systems mutually interact in the body to maintain individual vital organ function and physiologic homeostasis (Godin and Buchman, 1996). If organ systems are not able to coordinate appropriately, this disruption of inter-organ relationships is likely to be associated with death. In fact, multiple organ failure is the main cause of death in patients treated in intensive care units (ICUs) (Mayr et al., 2006; Orban et al., 2017). Consistently, life-threatening conditions like sepsis commonly affect multiple organ systems concurrently (Godin and Buchman, 1996; Bartsch et al., 2015; Asada et al., 2016, 2019). Using correlation-based network analyses of parameters representing main organ systems, Asada et al. (Asada et al., 2016) have shown that groups of critically ill ICU patients differing with respect to survival also had different network characteristics, focusing on a cross-sectional comparison of networks between a survivor and non-survivor group at ICU admission. We extend this work by including a longitudinal network comparison which, besides ICU admission, comprises an additional clinically relevant event time point to capture important network dynamics associated with survival: the 48–24 h period prior to death in non-survivors or the corresponding period after equal duration of ICU treatment in matched survivors (Figure 2). We hypothesize that survivor and non-survivor networks evolve differently in the course of ICU treatment.

#### Study Design, Data and Implementation Details

In our study, we consider the same nine different organ systems as in Asada et al. (2016), represented by *N* = 9 specific network parameters or nodes, respectively (Table 1). Partly in contrast to Asada et al. (2016), we here consider routine laboratory parameters and vital sign measurements that are regularly determined in the course of ICU treatment.

**Table 1:** Parameters representing the different organ systems in our study.

**Organ (system)**	**Parameter**	**Symbol**
Liver (hepatic)	Bilirubin	Bil
Neuroendocrine	Sodium	Na
Kidney (renal)	Creatinine	Cre
Immune system (inflammation)	C-reactive protein	CRP
(glucose) Metabolism	Blood glucose	Glu
Lung/respiration	Horovitz quotient (PF ratio)	PF
Hematopoiesis	Hemoglobin	Hb
Cardiovascular system	Mean arterial pressure ^*^	MAP
Coagulation/thrombosis	Platelet count ^*^	Plt

* *Parameters chosen differently compared to Asada et al. (2016)*.

We analyze consecutive admissions to the surgical ICU of the University Medical Centre Mannheim (Germany) of patients 18 years or older with a minimum length-of-stay of 72 h between 07/18/2016 and 11/04/2018. Follow-up of this cohort starts on ICU admission after beginning of the study period, and ends on ICU discharge, death or end of the study period, whichever is earliest. The study outcome is ICU mortality. For cases experiencing this outcome, we define the 24-h interval immediately after admission as the admission time window and the 48–24 h time window prior to the time of death as the event time window. We only retain cases with at least one recorded value for each of the network parameters (Table 1) in both admission and event time window, where values for bilirubin and the Horovitz quotient may have been imputed using specifically designed imputation schemes (Supplementary Material 1).

**Figure 3:**
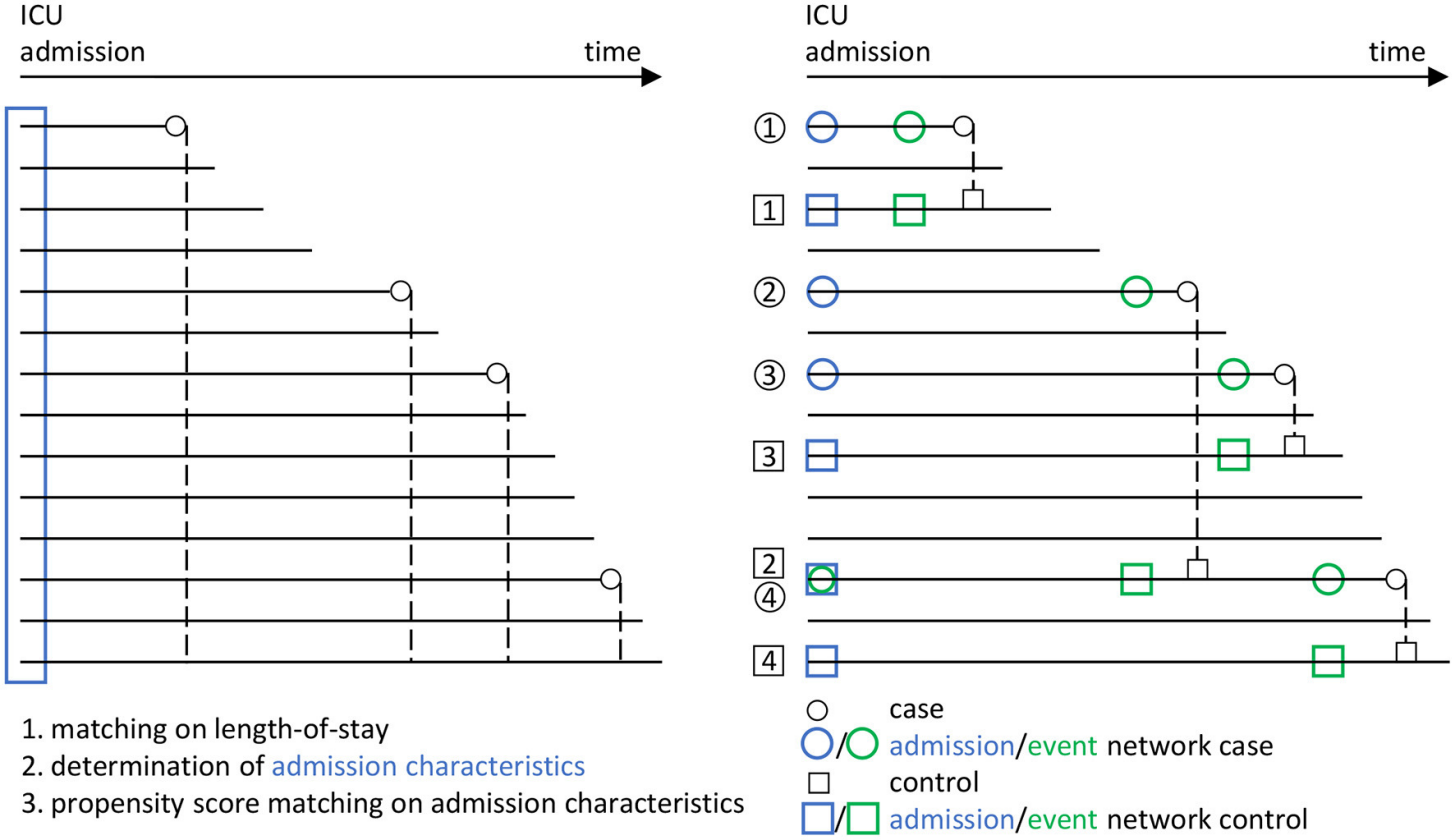
Overview of the matching procedure to identify appropriate controls (i.e., a survivor group) to the cases (i.e., the group of non-survivors from the ICU patient data base) in our study, using a combined risk set sampling (Langholz and Goldstein, 1996) and propensity score matching (Rosenbaum and Rubin, 1983) approach. In line with Figure 2, circles are used for cases and rectangles for controls. Blue color indicates the admission stage and green color the event stage. Each horizontal line represents an encounter in the ICU from the data base. For the cases, the corresponding death times are indicated by small circles. In a first step, a matching with respect to the ICU length-of-stay is performed, in that each potential control has to have at least the same length-of-stay than the corresponding case (dashed lines). In a second step, the admission characteristics of all encounters are determined (blue rectangle). These are then employed in a third step, in which a propensity score matching with respect to the admission characteristics is performed, to the end that the controls should have similar admission characteristics as the cases. Using this procedure, we end up with several case-control pairs (here, the pairs 1, 2, 3, and 4). In particular, we employ a nested case-control study design (Ernster, 1994; Keogh and Cox, 2014), in which an encounter may take the role of both a case and a control (see e.g., the encounter taking the role of a case in case-control pair 4, but the role of a control in case-control pair 2). Eventually, from the set of case-control pairs, the four different networks considered in our analyses (Figure 2) are derived.

Using risk set sampling (Langholz and Goldstein, 1996) we select propensity score and length-of-stay-matched controls as follows (Figure 3): For each case we identify as potential controls all ICU admissions of the cohort treated in the ICU for at least as long as the given case, independent of survival, corresponding to a nested case-control study design (Ernster, 1994; Keogh and Cox, 2014). This way, each risk set consists of a case and all admissions with matching on length-of-stay as controls, so that the sum of all controls by far exceeds the number of admissions. Each control's admission time window is determined as the 24-h interval after admission. We subsequently only consider controls with at least one recorded value for each of the network parameters in the admission time window. In each risk set, we calculate the index time for each remaining control as the sum of admission time and treatment duration of its corresponding case. We then determine the control's event time window as the 48–24 h time window prior to their index time. From each case's risk set, we then identify eligible controls with at least one recorded value of each of the network parameters in the event time window.

A propensity score (Rosenbaum and Rubin, 1983) for mortality risk based on data recorded closest to the admission time point during the admission time window is then determined for cases and controls. It is based on the admission simplified acute physiology score (SAPS) II (Le Gall et al., 1993) minus the points for age, systolic blood pressure, Horovitz quotient, sodium and bilirubin, the admission 10-item therapeutic intervention scoring system (TISS) (Beier et al., 2019), age, sex, the network parameters, Charlson comorbidity index (Charlson et al., 1987) and catecholamine therapy started up to 8 h after admission. In each risk set, we match the control with the smallest Mahalanobis distance calculated from the logarithm of the propensity score and the network parameters to each case. In the course of this, we account for the goodness of the propensity score matching by excluding matches with a propensity score outside the common support for the case (non-survivor) and control (survivor) groups (i.e., the largest interval containing propensity scores for subjects in both groups).

The above procedure yields matched case-control pairs for which the selected controls may stem from the same encounters. This may lead to potential bias resulting from underestimation of variability of the control admission network by including the same encounters as controls multiple times. We therefore compute the absolute differences Δ: = |ps_case_−ps_control_| in propensity scores ps_case_ and ps_control_ for all concerned case-control pairs, respectively, and only keep the pair with the smallest value of Δ in our study, while excluding the other pairs.

At this point, we explicitly recall that the matching based on the *admission* characteristics in order to select the controls is an essential part of our procedure and of crucial importance for validity of the results, which may differ when using other matching strategies (Discussion).

**Figure 4:**
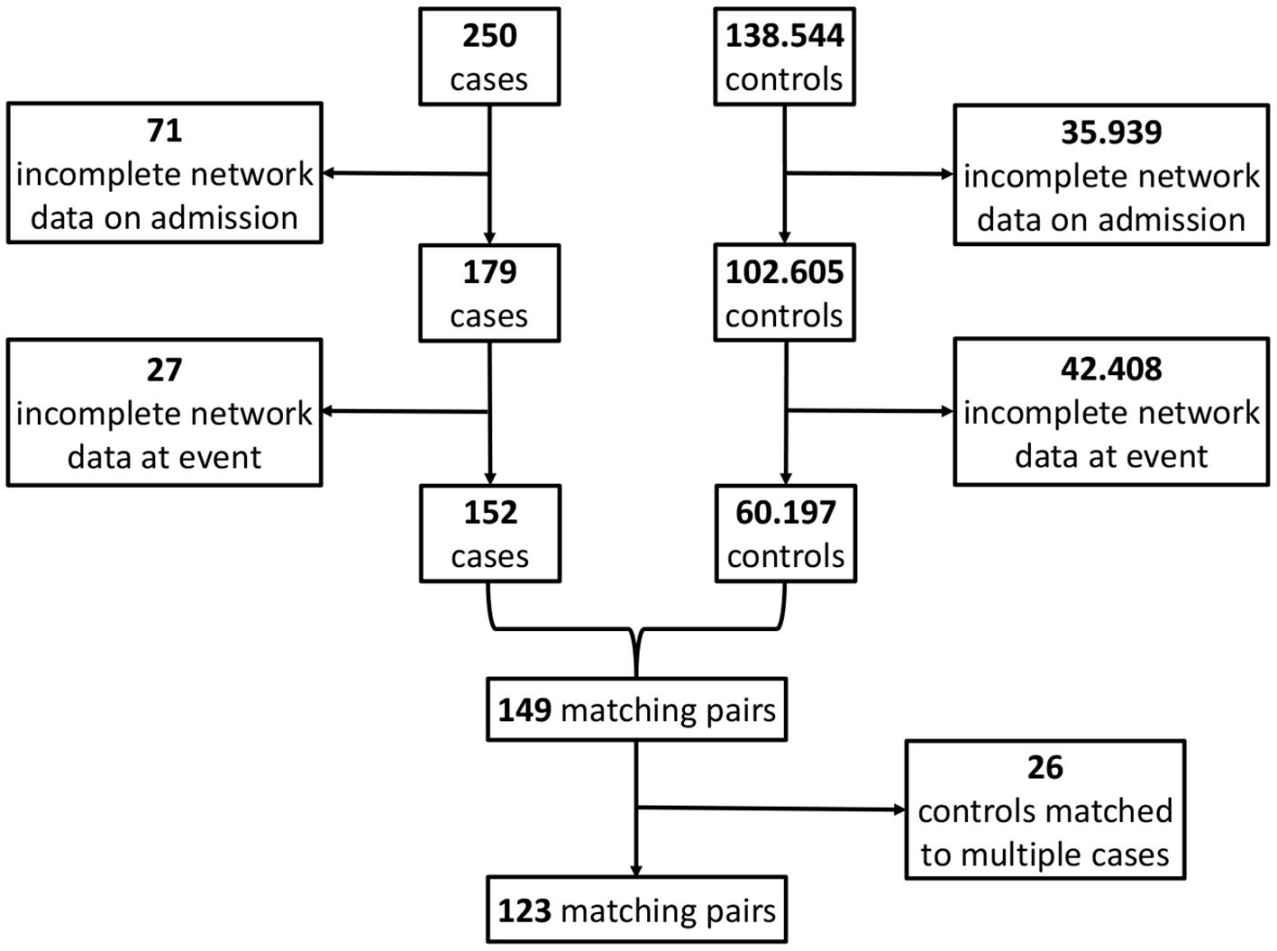
Flowchart for the matching procedure.

For the so-obtained *S* = 123 (Results; Figure 4) case-control pairs, we separately extract the values of the corresponding *N* = 9 network parameters for each of the four instances from Figure 2, i.e., (i) the non-survivors' admission, (ii) the survivors' admission, (iii) the non-survivors' event and (iv) the survivors' event network. Hence, for each of the four networks, we have an (*S* × *N*) data matrix as an input for network estimation.

Each of the network estimation approaches described before, in which edges are drawn based on different correlation concepts, is employed to derive the four networks. However, following Asada et al. (2016), our main focus is on networks estimated based on pairwise Spearman correlations between the nine parameters representing the organ systems. Here, an edge weight between two nodes is set to zero if the corresponding Bonferroni- or BH-adjusted *p*-value is > 0.05.

In our analyses, we consider the following four network comparisons (Figure 2):

(C1) non-survivors at admission vs. survivors at admission,
(C2) non-survivors at event vs. survivors at event,
(C3) survivors at admission vs. survivors at event, and
(C4) non-survivors at admission vs. non-survivors at event.

The cross-sectional comparisons between non-survivors and survivors (C1 and C2) are based on unpaired samples (as then, for both networks the data may come from different encounters), whereas the longitudinal comparisons between admission and event stage (C3 and C4) are based on paired samples (as then, for both networks the data comes from the same encounters).

For the analyses, we consider the network difference characteristics as stated before, where *G, E* and *C* have already been employed in Asada et al. (2016), while the others are additionally considered here, and use *M* = 10, 000 permutations to derive *p*-values. We declare a difference between two networks with respect to a fixed network difference characteristic to be significant if the corresponding *p*-value is ≤ 0.05.

## Results

By applying the matching procedure described before to our ICU patients, we end up with *S* = 123 matched case-control pairs (Figure 4) and thus a considerably larger sample size than the 40 pairs in Asada et al. (2016). Details about the characteristics of the considered patient groups can be found in Table 2 and Supplementary Table 1.

**Table 2:** Basic characteristics of the non-survivor and survivor patient groups at ICU admission and event stage, consisting of *S* = 123 patients each, considered in our study.

	**ADMISSION STAGE**			**EVENT STAGE**		
	**NON-SURVIVORS (*S* = 123) mean (SD) or s (% of S)**	**SURVIVORS (*S* = 123) mean (SD) or s (% of S)**	***p*-value**	**NON-SURVIVORS (*S* = 123) mean (SD) or s (% of S)**	**SURVIVORS (*S* = 123) mean (SD) or s (% of S)**	***p*-value**
DEMOGRAPHICS						
Men, *s*(%)	80	78	0.7902			
	(65.0%)	(63.4%)				
Age, yr	67.9	68.6	0.6999			
	(14.1)	(12.9)				
ICU length of stay, d	12.6	22.4	<0.0001			
	(12.8)	(17.9)				
PRE-EXISTING CONDITIONS						
Charlson comorbidity index	3.5	3.6	0.8193			
	(2.8)	(2.8)				
CLINICAL INTERVENTIONS						
Catecholamines, *s*(%)	94	94	1.0000	99	87	0.0748
	(76.4%)	(76.4%)		(80.5%)	(70.7%)	
Mechanical ventilation, *s*(%)	119	117	0.5185	118	97	<0.0001
	(96.7%)	(95.1%)		(95.9%)	(78.9%)	
Dialysis, *s*(%)	12	9	0.4936	30	26	0.5430
	(9.8%)	(7.3%)		(24.4%)	(21.1%)	
CLINICAL SCORES						
SAPS II	20.4	19.6	0.5553	23.1^a^	17.0	<0.0001
	(9.5)	(9.6)		(9.2)	(8.3)	
TISS-10	21.4	21.0	0.7181	20.0^a^	16.5	<0.0001
	(7.0)	(7.8)		(6.9)	(6.3)	
SOFA	10.9^b^	9.3^d^	0.0361	11.0^c^	7.4^d^	<0.0001
	(3.2)	(3.8)		(4.0)	(3.7)	
NETWORK PARAMETERS					
Bilirubin, mg/dl	1.01	0.85	0.2432	1.80	1.03	0.0372
	(1.22)	(0.95)		(3.47)	(2.18)	
Sodium, mmol/l	130.0	139.3	0.7252	145.3	142.8	0.0427
	(6.2)	(5.0)		(10.9)	(8.4)	
Creatinine, mg/dl	1.64	1.42	0.1428	1.80	1.15	<0.0001
	(1.28)	(1.03)		(1.37)	(0.76)	
CRP, mg/l	126.0	118.6	0.6140	163.5	123.6	0.0017
	(109.3)	(118.1)		(106.0)	(90.2)	
Blood glucose, mg/dl	130	134	0.7413	137.9	137.3	0.9143
	(63.2)	(45.3)		(39.5)	(34.9)	
Horovitz quotient, mmHg	351.4	336.0	0.5210	283.2	324.2	0.0066
	(199.0)	(176.3)		(113.6)	(120.9)	
Hemoglobin, g/dl	10.6	10.4	0.4062	9.1	9.2	0.6986
	(2.2)	(2.1)		(1.6)	(1.4)	
MAP, mmHg	82.1	81.3	0.6953	78.6	85.0	0.0039
	(15.2)	(14.7)		(17.2)	(17.4)	
platelet count, 10^9^/l	222.6	220.2	0.8805	211.0	270.2	0.0036
	(132.7)	(116.0)		(157.0)	(158.4)	

*Results are represented in the form mean (standard deviation, SD) for non-binary variables and s (% of S) for binary variables, with s ≤ S being a number of patients. P-values are derived from t-tests (continuous variables) or χ^2^-tests (categorical variables). SAPS II, simplified acute physiology score II; TISS-10, 10-item therapeutic intervention scoring system; SOFA, sequential organ failure assessment; CRP, C-reactive protein; MAP, mean arterial pressure. In case of missing data*:

a *based on S = 121 patients*;

b *S = 55*;

c *S = 53*;

d *S = 44*.

*Further details are given in Supplementary Table 1*.

Average age and proportion of male patients in non-survivors and matched survivors are generally compatible with ICU patients. A relatively large proportion of patients is mechanically ventilated or receives catecholamines, slightly less in the survivor group at the event stage. In line with the aim of our case-control matching procedure, at the ICU admission stage, there are no significant differences (at a 5% level) between the non-survivor and the survivor group with respect to practically all considered quantities. One exception is the sequential organ failure assessment (SOFA) score (*P* = 0.0361, Table 2), which was not included in the propensity score as its components are largely represented in the network parameters and other disease severity scores. The observed statistically significant difference in average SOFA scores on admission can be considered as being of marginal clinical significance.

At the event stage, meaningful differences between non-survivors and survivors in our study can be observed, in that the clinical scores (SAPS II, TISS-10, SOFA) are significantly higher for non-survivors compared to survivors, and the values of the nine considered network parameters indicate comparably worse clinical states for non-survivors.

Overall, the Spearman correlations between the network parameters for the four networks in our setting are basically rather weak. Significant (absolute) correlations are typically stronger at event stage than at admission stage (Supplementary Material 2.2, Supplementary Figures 1–4).

**Figure 5:**
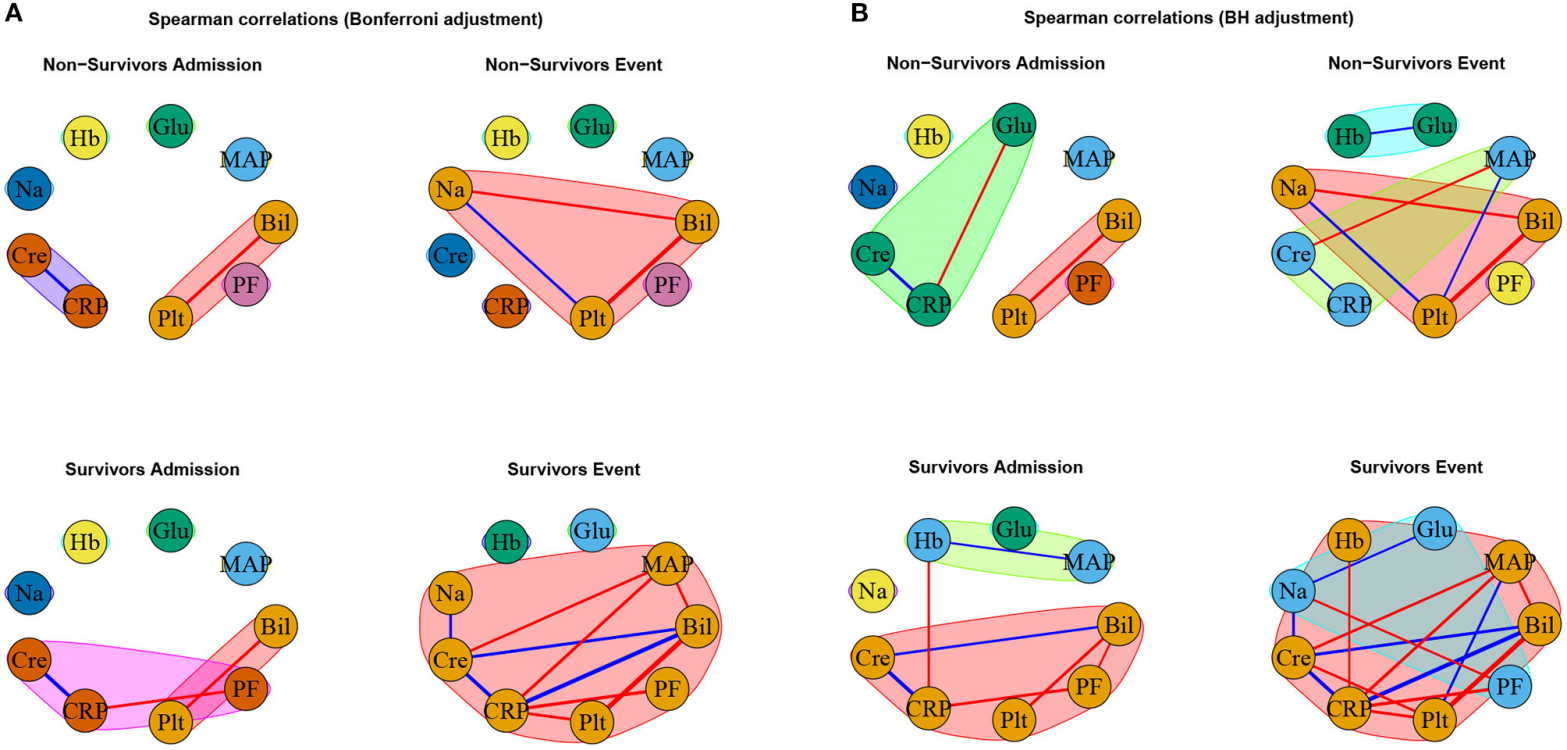
Networks estimated based on Spearman correlations together with **(A)** Bonferroni and **(B)** BH adjustment, respectively. Positive associations are indicated in blue, and negative associations in red. The thickness of the edges refers to the absolute magnitude of the correlation (the higher the correlation the thicker the edge).

The specific outcomes for our cross-sectional and longitudinal comparisons depend on the considered network estimation method, as well as on the considered network difference characteristic. As previously mentioned, we mainly limit our discussion to estimated networks based on Spearman correlations together with Bonferroni (Figure 5A and Table 3) or BH (Figure 5B and Table 4) adjustment in what follows, while the major observations also remain valid when considering alternative network estimation approaches based on distance correlation (Supplementary Material 2.3, Supplementary Figure 5, Supplementary Tables 2, 3) and EBICglasso (Supplementary Material 2.4, Supplementary Figures 6, 7, Supplementary Table 4), respectively.

**Table 3:** *P*-values corresponding to the cross-sectional and longitudinal comparisons between networks estimated using Spearman correlations together with Bonferroni adjustment (Figure 5A) for different network difference characteristics: global strength, Frobenius metric, maximum metric, spectral distance, Jaccard distance, number of edges, number of clusters, number of isolated nodes, degree of a specific node *i* (only nodes corresponding to a *P* ≤ 0.05 are shown), edge strength between two specific nodes *i* and *j* (only edges corresponding to a *P* ≤ 0.05 are shown).

		**Cross-sectional**		**Longitudinal**	
		**(C1) Non-survivors admission vs. Survivors admission**	**(C2) Non-survivors event vs. Survivors event**	**(C3) Survivors admission vs. Survivors event**	**(C4) Non-survivors admission vs. Non-survivors event**
Overall	Global strength	0.5183	**0.0414**	**0.0002**	0.5590
	Frobenius metric	0.8541	**0.0186**	**0.0225**	0.4175
	Maximum metric	0.7097	**0.0428**	0.0534	0.4576
	Spectral distance	0.7417	0.0851	**0.0010**	0.4273
	Jaccard distance	0.9281	**0.0087**	0.1790	0.5068
	Number of edges	0.7439	0.0661	**0.0009**	0.7870
	Number of clusters	0.6943	**0.0169**	0.0516	1.0000
	Number of isolated nodes	0.7840	**0.0365**	0.2913	0.8230
Nodes	Degree of node *i*	None	CRP: **0.0054**	Cre: **0.0116**	Plt: **0.0355**
				Na: **0.0176**	
				MAP: **0.0363**	
				Bil: **0.0363**	
				Plt: **0.0369**	
Edges	Edge strength between nodes *i* and *j*	None	Bil-CRP: **0.0001**	Bil-CRP: **<0.0001**	Na-Plt: **0.0086**
			Bil-Na: **0.0078**	CRP-Plt: **<0.0001**	
			Na-Plt: **0.0108**	Na-Cre: **0.0019**	
			CRP-Plt: **0.0194**	MAP-CRP: **0.0130**	
				Bil-Plt: **0.0383**	

*P ≤ 0.05 are indicated in bold font*.

**Table 4:** *P*-values corresponding to the cross-sectional and longitudinal comparisons between networks estimated using Spearman correlations together with BH adjustment (Figure 5B) for different network difference characteristics: global strength, Frobenius metric, maximum metric, spectral distance, Jaccard distance, number of edges, number of clusters, number of isolated nodes, degree of a specific node *i* (only nodes corresponding to a *P* ≤ 0.05 are shown), edge strength between two specific nodes *i* and *j* (only edges corresponding to a *P* ≤ 0.05 are shown).

		**Cross-sectional**		**Longitudinal**	
		**(C1) Non-survivors admission vs. Survivors admission**	**(C2) Non-survivors event vs. Survivors event**	**(C3) Survivors admission vs. Survivors event**	**(C4) Non-survivors admission vs. Non-survivors event**
Overall	Global strength	0.2757	**0.0474**	**0.0042**	0.2994
	Frobenius metric	0.6707	**0.0140**	**0.0023**	0.5166
	Maximum metric	0.6185	**0.0434**	**0.0237**	0.9003
	Spectral distance	0.4971	0.0886	**0.0046**	0.3318
	Jaccard distance	0.6502	**0.0287**	0.0670	0.6485
	Number of edges	0.3349	0.1107	**0.0340**	0.3260
	Number of clusters	0.5102	0.3605	0.3849	0.5487
	Number of isolated nodes	0.5962	0.8208	0.3194	0.4246
Nodes	Degree of node *i*	None	CRP: **0.0217**	Plt: **0.0007**	Plt: **0.0078**
				Na: **0.0017**	MAP: **0.0455**
Edges	Edge strength between nodes *i* and *j*	None	Bil-CRP: **0.0001**	Bil-CRP: **<0.0001**	Na-Plt: **0.0086**
			Bil-Na: **0.0078**	CRP-Plt: **<0.0001**	MAP-Plt: **0.0099**
			Na-Plt: **0.0108**	Na-Cre: **0.0019**	
			CRP-Plt: **0.0194**	Glu-Na: **0.0074**	
				MAP-CRP: **0.0130**	
				Cre-Plt: **0.0214**	
				MAP-Plt: **0.0232**	
				Bil-Plt: **0.0383**	
				Na-PF: **0.0426**	

*P ≤ 0.05 are indicated in bold font*.

As expected, for the Spearman correlation networks based on the more conservative Bonferroni adjustment (Figure 5A), fewer edges can be observed as for those based on the more progressive BH adjustment (Figure 5B).

When comparing the networks for non-survivors and survivors at ICU admission (C1), no significant differences can be observed for all considered network difference characteristics, with the networks showing a similar structure. This is to be expected, as the matching procedure we employed for defining the non-survivor and survivor group followed the principle of making admission characteristics as similar as possible.

In contrast, when comparing the networks for non-survivors and survivors at the event stage (C2), C-reactive protein (CRP) shows a significantly different degree. Specifically, CRP has no or one edge, respectively, in the non-survivor event network, but is highly connected with many edges and a high degree in the survivor event network (*p*-values: *P* = 0.0054, Table 3, and *P* = 0.0217, Table 4). Further, the positive association between bilirubin and CRP (*P* = 0.0001, Tables 3, 4) is significantly different, in that it is present in the survivor group, but not in the non-survivor group. Conversely, the negative association between bilirubin and sodium is present in the non-survivor group, but absent in the survivor group (*P* = 0.0078, Tables 3, 4). A similar pattern (as for bilirubin) in the interplay with CRP and sodium can be observed for platelet count: A negative association between platelet count and CRP is present in the survivor group and absent in the non-survivor group (*P* = 0.0194, Tables 3, 4), while the converse holds for the positive association between platelet count and sodium (*P* = 0.0108, Tables 3, 4). Significant differences also exist with respect to several overall network difference characteristics (Tables 3, 4). In particular, in case of the Bonferroni adjustment, there are many edges and few clusters in the survivor event network (*P* = 0.0169 for differences in the number of clusters, Table 3) and many isolated parameters with no edges in the non-survivor event network (*P* = 0.0365 for differences in the number of isolated nodes, Table 3). However, for the BH adjustment, there are in contrast no significant differences with respect to the number of clusters and isolated nodes for comparison (C2) (Table 4). This is due to the higher connectivity of the event networks in case of the BH adjustment, compared to that obtained using the more conservative Bonferroni adjustment (Figure 5).

Regarding the longitudinal comparison between admission and event stage for the survivor group (C3), sodium (*P* = 0.0176, Table 3, and *P* = 0.0017, Table 4) and platelet count (*P* = 0.0369, Table 3, and *P* = 0.0007, Table 4) show a significantly different degree for both adjustment methods. Moreover, for instance, the positive association between bilirubin and CRP (*P* < 0.0001, Tables 3, 4), the negative association between CRP and platelet count (*P* < 0.0001, Tables 3, 4) and the positive association between sodium and creatinine (*P* = 0.0019, Tables 3, 4) are present at the event stage only and not at admission. Significant differences for survivors between admission and event stage can also be observed with respect to several overall network difference characteristics (Tables 3, 4).

Finally, the comparison between admission and event stage for the non-survivors (C4) reveals no significant differences for all overall network difference characteristics. platelet count have significantly more edges at event stage than at admission (*P* = 0.0355, Table 3, and *P* = 0.0078, Table 4), and the positive association between sodium and platelet count, being only present at the event stage, is significantly different between the two networks (*P* = 0.0086, Tables 3, 4).

Overall, based on the above results for the comparisons (C1) to (C4), we observe diverging dynamics between survivors and non-survivors in the course of the ICU stay.

Interestingly, the negative association between bilirubin and platelet count forms the only edge that is present in all networks (Figures 5A,B).

To investigate the effect of medical intervention on the results, we exemplarily consider renal replacement therapy (RRT; dialysis) as one of the most frequent therapies and additionally re-perform our analyses for the subgroup of encounters that did not receive RRT at admission or event stage (Supplementary Material 3, Supplementary Figures 8–12, Supplementary Tables 5, 6). In terms of the overall network difference characteristics, the results of the analyses basically continue to hold for this subgroup of encounters, thus showing that the intervention by an RRT has only a minor influence on the analyses of overall network structures, if any. However, partly different results are obtained with respect to the edge-specific difference in edge strength: On the one hand, the results are still similar for the comparisons (C1) and (C3). On the other hand, for the comparisons (C2) and (C4), all edges that are found to be significantly different for the overall patient group are not significantly different anymore for the subgroup of encounters not receiving RRT. Most prominently, for the overall patient group, a positive association between sodium and platelet count is only present in the non-survivor event network, yielding significant differences with respect to edge strength for the comparisons (C2) (*P* = 0.0108, Tables 3, 4) and (C4) (*P* = 0.0086, Tables 3, 4). In the subgroup of patients not receiving RRT, the sodium-platelet count association vanishes, and so do the respective significant differences for (C2) and (C4) (Supplementary Material 3).

## Discussion

We have presented an overall framework for differential network comparison, with several options for both the network estimation and the network characteristic according to which a difference is measured, and implemented it in the R package DNT. To test for differences between two networks, we use a permutation-based procedure, which is generally applicable, but typically comes with increased computational running time compared to specifically tailored tests based on asymptotic theory. To reduce computing time in our R package, the (repeated) calculations related to a permutation test may in the future be outsourced to computationally faster C++ implementations, as for instance performed similarly in Schefzik et al. (2021).

Besides the established network estimation methods presented here, one could in a future work also consider other, more recent options, e.g., based on the distance precision matrix that can take account of non-linear relationships (Ghanbari et al., 2019) or the new coefficient of correlation introduced in Chatterjee (2020). Similarly, alternative, more complex network difference characteristics than those considered here may be based, e.g., on centrality measures in general (Oldham et al., 2019) or community-based measures (Chen et al., 2013; Labatut, 2015; Gupta et al., 2016; Ghalmane et al., 2019), or may be specifically tailored to the comparison of networks consisting of different nodes (Tantardini et al., 2019).

In our application, we have demonstrated that network comparisons reveal insights into the dynamics of organ system interactions in intensive care patients. Particularly, while the corresponding networks for a survivor and a non-survivor patient group are not significantly different at ICU admission, they evolve differently in the course of the ICU stay, in that significantly more network edges (organ system interactions) at the event stage can be observed for the survivors, but not for the non-survivors. A possible interpretation for typically few organ system interactions on ICU admission may be similar acute medical conditions in both patient groups (comparison C1). During the ICU stay, significant stabilization in survivors may lead to more organ system interactions for this group at the event stage compared to the admission stage (C3). In contrast, non-survivors fail to significantly stabilize at the event stage (prior to death) compared to the admission stage (C4), potentially due to the inability to regain coordination between organs during the ICU stay or unsuccessful therapy. Hence, significantly different network structures at the event stage can be observed between the survivor and the non-survivor group (C2).

Besides the overall network dynamics discussed above, our analyses also reveal insights into the role of and the correlations between specific parameters.

For instance, we observe a comparably strong positive association between bilirubin and CRP in the survivors' event network only, but not in the non-survivors' event and the survivors' admission networks, leading to clearly significant differences in comparisons (C2) and (C3), respectively. CRP is primarily synthesized in the liver (Sproston and Ashworth, 2018), and bilirubin represents the hepatic system in our network. Their common organ source may play a role for the observed association. Throughout, CRP values in our ICU patients are above the reference range for healthy individuals. In the non-survivors' event network, we observe a U-shaped relationship between bilirubin and CRP: On the one hand, lower CRP values of up to around 100 mg/l are typically linked to higher bilirubin values (negative correlation). This agrees with frequently impaired CRP production in liver failure with increased bilirubin values (Sproston and Ashworth, 2018). On the other hand, there is a positive correlation between higher CRP values (above around 100 mg/l) and bilirubin. In summary, these opposite correlations largely cancel each other out, leading to an overall Spearman correlation of 0.01 (i.e., virtually zero; Supplementary Figure 3). In contrast, for the survivors' event network, bilirubin and CRP overall show a stronger positive Spearman correlation of 0.47 (Supplementary Figure 4). At the event stage, both bilirubin and CRP values alike are significantly lower for the survivors than for the non-survivors, while at the admission stage, there is no significant cross-sectional difference (Table 2). The latter, however, may at least partly be attributable to our matching strategy. All in all, our findings support the statement in Lippi and Targher (2012) that there may be “the possibility of a more complex biological interaction between degree of the inflammatory state and bilirubin metabolism that merits further investigation” (Lippi and Targher, 2012, p. 2230).

Moreover, the negative association between bilirubin and platelet count forms the only edge that is present in all networks. Indeed, it reveals the second strongest (absolute) correlation in the survivors' admission network and the strongest in all other considered networks (Supplementary Figures 1–4). This is not unexpected, as thrombocytopenia is common in acute liver failure without being accounted for by hemorrhagic complications, and as its progression is positively correlated with the degree of hepatic encephalopathy, vasopressor requirement and RRT, and is associated with death or liver transplantation (Scharf, 2021). A reduction of hepatic synthesis of thrombopoietin, and thus thrombocytopoiesis, may account for the negative association between bilirubin and platelet count (Scharf, 2021).

Notably, we observe significantly more associations for CRP than for other parameters in the survivors' event network compared to the non-survivors' event network. CRP is a rather unspecific marker of inflammation and is likely to be associated with most of our considered network parameters (such as creatinine, bilirubin, mean arterial pressure and the Horovitz quotient; as seen for the survivors' event network) and respective organ systems (Table 1). We hypothesize that the low connectivity of CRP in non-survivors is overall reflective of failing organ system coordination prior to death.

Despite our observed network dynamics, the correlations underlying our analyses are arguably rather weak (Supplementary Material 2.2, Supplementary Figures 1–4). This may be due to our specific setting of critically ill patients, for which organ system interactions may be limited anyway. More specifically, for this patient group, an absence of variability, which is characteristic for disease states (Godin and Buchman, 1996), can be expected, and can explain the weak correlations.

The deceased critically ill patients and the matched survivors with very similar characteristics on admission can be considered as a typical sample of patients from a tertiary referral center's surgical ICU exemplified by a large proportion of neurosurgical admissions and patients admitted with sepsis (Supplementary Table 1). Their disease severity, however, is relatively high compared to patients usually treated in this setting. Moreover, due to our study design our study population is restricted to patients with a minimum ICU length-of-stay of 72 h, thus excluding non-survivors with short ICU stays.

In our analyses, specific results depend on the considered network estimation method and the network difference characteristic. We think it is therefore advisable to consider several methods concomitantly and to draw general conclusions based on this.

Moreover, the chosen matching procedure for control selection in the study may also critically affect the results of the analyses. We here perform a matching based on the admission characteristics, while removing possible duplicate controls according to an optimality criterion based on propensity scores. We choose this approach, as starting with cases and controls that are as similar as possible at admission stage appears to be most suitable to investigate our key question, i.e., whether non-survivors and survivors show different correlation network dynamics during the ICU stay. Indeed, the matching based on admission characteristics works quite well, witnessed by the fact that there are widely non-significant differences between non-survivors and survivors at ICU admission with respect to the considered quantities (Table 2 and Supplementary Table 1). However, other matching strategies may be possible and reasonable as well and might lead to an alternative selection of controls. Therefore, we additionally compare our findings to those obtained using a repeated random selection of controls (Supplementary Material 4, Supplementary Figures 13–19). We thereby both illustrate our specific, propensity score-based control selection method and confirm the validity of our approach.

Further, we recall that in our case-control matching procedure (Figure 3), cases may serve as controls prior to becoming a case. Duplicates occurring this way are retained in our analysis, which may potentially bias the results of the comparison of the admission networks between the survivor and the non-survivor group (C1) to the null. In our analysis, 18 out of the 123 cases (14.6%) indeed also serve as a control. However, this does not actually compromise our results for comparison (C1) here. Specifically, when removing the corresponding 18 case-control pairs and re-performing the analysis for (C1), there are no significant differences with respect to all considered network difference characteristics (results not shown).

As the event time point in our study, we consider the 48–24 h period prior to death for the non-survivors, and a corresponding matching time point accounting for the ICU length-of-stay for the survivors, respectively. This appears to be reasonable because in intensive care, therapy withdrawal usually only occurs shortly before death. Therefore, it is rather unlikely that for the non-survivors, essential life-sustaining medical interventions are stopped during the time frame of 48–24 h prior to death. Consequently, the probability of receiving interventions affecting their network parameters likely remains comparable to those of survivors. In contrast, decease-related changes in network structure might already be evident at this time stage.

We emphasize again that in our study, the term "admission" refers to a time frame that is equal during the ICU stay for all included patients, namely the 0–24 h interval after ICU admission. In contrast, this does not hold for the term "event," as the time from ICU admission to the event time point of 48–24 h prior to death (or the corresponding matching time point for survivors) can vary from patient to patient. Consequently, we currently do not consider fixed treatment lengths and thus do not distinguish between a comparably early or late decease. Future adaptations of our study may deal with adequately incorporating a respective time dependency.

In the future, additional organ systems could be included in the study. Alternatively, instead of specific organ systems, numerous clinical or laboratory parameters could be considered concurrently but detached from the concept of organ systems.

In the presented study, the outcome is ICU mortality. However, other clinically relevant outcomes such as development of sepsis could also be investigated.

Currently, our results provide pathophysiological and pathomechanistic insights on a global patient group basis. In particular, the liver appears to play a central role in our analyses, in that those network parameters that are involved in the most prominent observations as discussed before (bilirubin, CRP, platelet count) are directly related to the liver (function). Hence, we hypothesize that an intensive monitoring of the liver during ICU stay is crucial and that considering additional liver parameters (Kasarala and Tillmann, 2016) may be beneficial.

However, at this point, it appears to be challenging to transfer our group-based results to an individual patient level. For instance, it is not clear whether or to which extent our results can be used for a prediction of the course of an ICU stay for a specific patient already at ICU admission stage. Future research into this direction (Haslbeck and Waldorp, 2018) is strongly encouraged.

## Conclusion

We have introduced a framework for testing for differential networks, implemented in our R package DNT and comprising various options for network estimation and network difference characteristics. In an application to patient data from an ICU, cross-sectional and longitudinal network comparisons reveal diverging dynamics of organ system interactions between a survivor and non-survivor group of critically ill patients in the course of ICU treatment. The liver appears to play a central role for the observed increased connectivity in the survivor network at the event stage.

## Data Availability Statement

The R package DNT and the associated R Shiny application are freely available at https://github.com/RomanSchefzik/DNT. In the same repository, the data tables based on which our network comparison analyses have been performed are provided.

## Ethics Statement

The studies involving human participants were reviewed and approved by the Medical Ethics Commission II of the Medical Faculty Mannheim, Heidelberg University (2016-840R-MA). Written informed consent for participation was not required for this study in accordance with the national legislation and the institutional requirements.

## Author Contributions

RS, HL, and VS-L: methodology and study conceptualization and writing and editing. BH: data preparation. RS and BH: analyses. RS and LB: package programming. RS, TK, HL, MT, and VS-L: interpretation and discussion of results. RS, BH, and VS-L: figures. All authors contributed to the article and approved the submitted version.

## Funding

The work has been funded by the Klaus Tschira Foundation through the SCIDATOS project.

## Conflict of Interest

The authors declare that the research was conducted in the absence of any commercial or financial relationships that could be construed as a potential conflict of interest.

## Publisher's Note

All claims expressed in this article are solely those of the authors and do not necessarily represent those of their affiliated organizations, or those of the publisher, the editors and the reviewers. Any product that may be evaluated in this article, or claim that may be made by its manufacturer, is not guaranteed or endorsed by the publisher.
