# Supplementary material for: Differential Network Testing Reveals Diverging Dynamics of Organ System Interactions for Survivors and Non-survivors in Intensive Care Medicine
Source: Front Physiol. 2022 Jan 10;12:801622. doi: 10.3389/fphys.2021.801622 (PMC8784681; doi:10.3389/fphys.2021.801622)
Supplement: Supplementary file 1 [file Data_Sheet_1.PDF]

Supplementary Material to

Differential network testing reveals diverging dynamics of organ system interactions for survivors and non-survivors in intensive care medicine

Roman Schefzik, Leonie Boland, Bianka Hahn, Thomas Kirschning, Holger A. Lindner, Manfred Thiel and Verena Schneider-Lindner

S1 Supplementary methods: Imputation strategies

In our raw intensive care unit (ICU) patient data set, bilirubin and the Horovitz quotient (more specifically, the fraction of oxygen in the inhaled air, FiO_2, that is needed to derive the Horovitz quotient) turn out to be the network parameters for which values were missing most frequently. Thus, specific imputation strategies for these parameters are implemented in order not to lose too much data. For other network parameters, no imputation is performed.

S1.1 Imputation strategy for bilirubin

Bilirubin values may be often missing, as they are typically only determined in case of a corresponding clinical suspicion. In case a bilirubin value is missing at ICU admission, it can be expected to become available from routine laboratory analyses at the latest within one week. If this corresponding bilirubin value is within the reference interval, it may be used as the admission value. If a bilirubin value is abnormal some days after ICU admission, it is rather likely that the value has been within the reference interval before. Based on this rationale, we impute missing bilirubin values as follows:

- If a bilirubin value is missing at ICU admission,
  - we take the closest value from the past from up to 7.5 days before ICU admission, if available.
  - and if a bilirubin value from the past is not available, we consider the closest value from the future from up to 7.5 days after ICU admission:
    * If the future bilirubin value is within the reference interval 0.1-1.0 (mg/dl), we take this value.
    * If the future bilirubin value is > 1.0, we draw a random value between 0.8 and 1.0 and take this value.

    Here, we only consider values associated to time points before the event (cases) or the matching event time point (controls).
- If a bilirubin value is missing in the course of the ICU stay, we take the closest value from the past from up to 7.5 days before the time point of interest, if available. In particular, no imputation based on future values is performed.
- If no bilirubin value can be determined using the above procedure(s), we draw a random value between 0.2 and 1.0 and take this value.

S1.2 Imputation strategy for the Horovitz quotient

The Horovitz quotient is defined as the ratio of partial pressure of oxygen in blood (PaO_2; mmHg) and the fraction of oxygen in the inhaled air (FiO_2). In our data, missing FiO_2 values are imputed as follows:

- Whenever possible, we take a measured FiO_2 value.
- If a measured FiO_2 value is missing, we use a corresponding set FiO_2 value, if available.
- If both a measured and a set FiO_2 value are missing, we proceed as follows:
  - If the patient is mechanically ventilated, we take an FiO_2 value of 0.3.
  - If the patient is not mechanically ventilated, we take an FiO_2 value of 0.21 (ambient air).

Moreover, during the interval from two hours prior to the start of a lung replacement therapy (ECMO/PECLA) until one hour after the end of the therapy, the Horovitz quotients is set to missing, and the corresponding cases are consequently excluded from our analyses, which require complete data.

S2 Supplementary results

S2.1 Basic patient characteristics

Table S1: Basic characteristics of the non-survivor and survivor patient groups at ICU admission and event stage, consisting of $S = 123$ patients each, considered in our study. Results are represented in the form median (interquartile range, IQR) and mean (standard deviation, SD) for non-binary variables and s (% of S) for binary variables, with $s \leq S$ being a number of patients. P-values are derived from t-tests (continuous variables) or χ^2-tests (categorical variables); *: p-values derived from Fisher's exact tests (categorical variables). SAPS II: simplified acute physiology score II; TISS-10: 10-item therapeutic intervention scoring system; SOFA: sequential organ failure assessment; CRP: C-reactive protein; MAP: mean arterial pressure, pO_2: oxygen partial pressure; pCO_2: carbon dioxide partial pressure; FiO_2: fraction of inspired oxygen; aPTT: activated partial thromboplastin time. In case of missing data: ^{a}: based on $S = 122$ patients; ^{b}: $S = 121$; ^{c}: $S = 120$; ^{d}: $S = 119$; ^{e}: $S = 118$; ^{f}: $S = 117$; ^{g}: $S = 116$; ^{h}: $S = 115$; ^{i}: $S = 114$; ^{j}: $S = 113$; ^{k}: $S = 111$; ^{l}: $S = 98$; ^{m}: $S = 68$; ^{n}: $S = 61$; ^{o}: $S = 60$; ^{p}: $S = 59$; ^{q}: $S = 55$; ^{r}: $S = 53$; ^{s}: $S = 47$; ^{t}: $S = 44$; ^{u}: $S = 43$, ^{v}: $S = 42$, ^{w}: $S = 36$.

	ADMISSION STAGE					EVENT STAGE				
	NON-SURVIVORS ($S = 123$)		SURVIVORS ($S = 123$)			NON-SURVIVORS ($S = 123$)		SURVIVORS ($S = 123$)		
	median (IQR) or s (% of S)	mean (SD)	median (IQR) or s (% of S)	mean (SD)	p-value	median (IQR) or s (% of S)	mean (SD)	median (IQR) or s (% of S)	mean (SD)	p-value
DEMOGRAPHICS										
men, s(%)	80 (65.0%)		78 (63.4%)		0.7902					
age, yr	70 (59-78)	67.9 (14.1)	71 (61-78)	68.6 (12.9)	0.6999					
ICU length of stay, d	8.8 (5.2-14.1)	12.6 (12.8)	16.3 (9.8-27.2)	22.4 (17.9)	<0.0001					
PRE-EXISTING CONDITIONS										
Charlson comorbidity index	3 (2-5)	3.5 (2.8)	3 (1-5)	3.6 (2.8)	0.8193					
diabetes, s(%)	27 (22.0%)		33 (26.8%)		0.3730					
respiratory diseases, s(%)	16 (13.0%)		18 (14.6%)		0.7118					
alcoholism, s(%)	4 (3.3%)		5 (4.1%)		1.0000*					
cardiovascular diseases, s(%)	79 (64.2%)		68 (55.3%)		0.1527					
sepsis on admission, s(%)	63 (51.2%)		51 (41.5%)		0.1250					
CLINICAL INTERVENTIONS										
catecholamines, s(%)	94 (76.4%)		94 (76.4%)		1.0000	99 (80.5%)		87 (70.7%)		0.0748
mechanical ventilation, s(%)	119 (96.7%)		117 (95.1%)		0.5185	118 (95.9%)		97 (78.9%)		<0.0001
dialysis, s(%)	12 (9.8%)		9 (7.3%)		0.4936	30 (24.4%)		26 (21.1%)		0.5430
blood transfusion (erythrocyte concentrate), s(%)	30 (24.4%)		29 (23.6%)		0.8813	24 (19.5%)		20 (16.3%)		0.5058
CLINICAL SCORES										
SAPS II	19 (13-27)	20.4 (9.5)	19 (10-25)	19.6 (9.6)	0.5553	23^b (16-28)	23.1^b (9.2)	16 (10-21)	17.0 (8.3)	<0.0001
TISS-10	22 (15-27)	21.4 (7.0)	22 (15-27)	21.0 (7.8)	0.7181	21^b (15-26)	20.0^b (6.9)	18 (10-21)	16.5 (6.3)	<0.0001
SOFA	11^q (9-13)	10.9^q (3.2)	10^t (6.5-12)	9.3^t (3.8)	0.0361	10^r (8-14)	11.0^r (4.0)	8^t (4-10.5)	7.4^t (3.7)	<0.0001

Table S1 continued

	ADMISSION STAGE					EVENT STAGE				
	NON-SURVIVORS ($S = 123$)		SURVIVORS ($S = 123$)			NON-SURVIVORS ($S = 123$)		SURVIVORS ($S = 123$)		
	median (IQR) or s (% of S)	mean (SD)	median (IQR) or s (% of S)	mean (SD)	p-value	median (IQR) or s (% of S)	mean (SD)	median (IQR) or s (% of S)	mean (SD)	p-value
NETWORK PARAMETERS										
bilirubin, mg/dl	0.63 (0.39-1.10)	1.01 (1.22)	0.57 (0.38-0.87)	0.85 (0.95)	0.2432	0.64 (0.36-1.62)	1.80 (3.47)	0.44 (0.29-0.74)	1.03 (2.18)	0.0372
sodium, mmol/l	139 (135-142)	130.0 (6.2)	139 (136-142)	139.3 (5.0)	0.7252	145 (136-151)	145.3 (10.9)	141 (137-148)	142.8 (8.4)	0.0427
creatinine, mg/dl	1.21 (0.77-2.04)	1.64 (1.28)	1.11 (0.78-1.72)	1.42 (1.03)	0.1428	1.39 (0.75-2.37)	1.80 (1.37)	0.89 (0.63-1.47)	1.15 (0.76)	<0.0001
CRP, mg/l	93.7 (39-208)	126.0 (109.3)	94.5 (14.7-181.0)	118.6 (118.1)	0.6140	131 (85.6-238.0)	163.5 (106.0)	116 (46.9-174.0)	123.6 (90.2)	0.0017
blood glucose, mg/dl	130 (103-169)	142.9 (63.2)	134 (109-154)	140.5 (45.3)	0.7413	134 (112-158)	137.9 (39.5)	135 (120-152)	137.3 (34.9)	0.9143
Horovitz quotient, mmHg	322.2 (197.1-457.1)	351.4 (199.0)	290.0 (217.0-414.3)	336.0 (176.3)	0.5210	271.3 (207.0-356.7)	283.2 (113.6)	309.3 (246.4-390.5)	324.2 (120.9)	0.0066
hemoglobin, g/dl	10.1 (8.9-12.0)	10.6 (2.2)	10.2 (8.7-11.6)	10.4 (2.1)	0.4062	8.9 (7.9-10.0)	9.1 (1.6)	8.9 (8.3-9.9)	9.2 (1.4)	0.6986
MAP, mmHg	80 (73-89)	82.1 (15.2)	78 (71-92)	81.3 (14.7)	0.6953	75 (65-90)	78.6 (17.2)	80 (72-94)	85.0 (17.4)	0.0039
platelet counts, 10^9/l	206 (133-278)	222.6 (132.7)	201 (150-281)	220.2 (116.0)	0.8805	177 (107-285)	211.0 (157.0)	234 (163-358)	270.2 (158.4)	0.0036
REFERRING DEPARTMENT										
anaesthesiology, s(%)	12 (9.8%)		5 (4.1%)		0.0785					
general surgery, s(%)	41 (33.3%)		38 (30.9%)		0.6821					
gynaecology, s(%)	0 (0%)		1 (0.8%)		1.0000*					
neuroradiology, s(%)	1 (0.8%)		2 (1.6%)		1.0000*					
neurosurgery, s(%)	44 (35.8%)		44 (35.8%)		1.0000					
orthopaedics/trauma centre, s(%)	11 (8.9%)		20 (16.3%)		0.0838					
otorhinolaryngology, s(%)	5 (4.1%)		5 (4.1%)		1.000					
radiology, s(%)	2 (1.6%)		0 (0%)		0.4980*					
urology, s(%)	6 (4.9%)		6 (4.9%)		1.0000					
other, s(%)	1 (0.8%)		2 (1.6%)		1.0000*					
BLOOD GAS ANALYSIS										
pH	7.356 (7.286-7.415)	7.348 (0.10)	7.368 (7.297-7.415)	7.356 (0.10)	0.5299	7.387 (7.299-7.446)	7.371 (0.09)	7.432 (7.397-7.462)	7.424 (0.05)	<0.0001
pO_2, mmHg	109 (84.1-135)	117.4 (45.0)	105 (78.6-138)	119.6 (64.9)	0.7536	89.6[a] (77.5-110)	96.7[a] (26.7)	92.8[b] (81.2-108)	99.9[b] (31.1)	0.3859
pCO_2, mmHg	42.6 (37.9-49.6)	45.2 (12.9)	43.7 (37.8-50.4)	45.1 (12.1)	0.9736	42.7[a] (35.2-49.6)	44.1[a] (13.6)	38.9[b] (36.6-44.0)	41.1[b] (8.0)	0.0371
bicarbonate, mmol/l	23.8 (20.4-26.4)	23.9 (4.7)	24.8 (21.9-26.7)	24.4 (4.3)	0.4356	24.1 (21.2-27.8)	24.6 (5.3)	26.6 (24.1-28.5)	26.4 (3.8)	0.0027
base excess, mmol/l	-1.1 (-4.8-2)	-1.1 (5.1)	-0.4 (-3.4-2.3)	-0.5 (4.7)	0.3998	-0.4 (-3.4-2.8)	-0.1 (5.4)	2.8 (-0.3-4.2)	2.3 (3.6)	<0.0001

Table S1 continued

	ADMISSION STAGE					EVENT STAGE				
	NON-SURVIVORS (S = 123)		SURVIVORS (S = 123)			NON-SURVIVORS (S = 123)		SURVIVORS (S = 123)		
	median (IQR) or s (% of S)	mean (SD)	median (IQR) or s (% of S)	mean (SD)	p-value	median (IQR) or s (% of S)	mean (SD)	median (IQR) or s (% of S)	mean (SD)	p-value
VITAL SIGNS										
systolic blood pressure, mmHg	118	120.8	118	120.8	0.9978	121	123.3	123	129.4	0.0622
	(107-131)	(23.0)	(104-134)	(22.4)		(108-136)	(25.1)	(110-142)	(26.2)	
diastolic blood pressure, mmHg	63	63.5	61	62.6	0.6185	56	59.6	59	61.9	0.2946
	(55-70)	(14.7)	(53-70)	(13.2)		(48-69)	(20.9)	(52-73)	(14.0)	
heart rate, 1/min	90	90.2	86	90.7	0.8762	94	96.6	85	85.7	$<$0.0001
	(72-105)	(23.2)	(71-107)	(26.5)		(81-114)	(22.4)	(72-100)	(20.2)	
shock index	0.73	0.78	0.74	0.78	0.8749	0.79	0.82	0.70	0.69	$<$0.0001
	(0.57-0.94)	(0.27)	(0.56-0.93)	(0.30)		(0.61-0.99)	(0.27)	(0.54-0.80)	(0.22)	
body temperature, $^{\circ}$C	36.3	36.2	36.5	36.5	0.0297	36.9[a]	37.0[a]	37.1[a]	37.2[a]	0.2313
	(35.6-37.0)	(1.2)	(36.0-37.3)	(1.3)		(36.3-37.7)	(1.1)	(36.7-37.6)	(0.7)	
volume balance, l	1.28	3.31	1.47	2.78	0.4715	0.79	1.58	0.15	0.48	0.0007
	(-0.02-4.01)	(6.45)	(-0.23-3.82)	(5.02)		(-0.03-2.22)	(3.05)	(-0.50-0.95)	(1.79)	
FiO_2, %	45	48.3	45	47.3	0.6802	30	37.8	30	30.7	$<$0.0001
	(35-60)	(18.2)	(35-60)	(18.6)		(30-40)	(14.8)	(22-35)	(8.2)	
expired minute volume, l/min	7.8[d]	7.7[d]	7.6[g]	8.1[g]	0.3758	9.3[e]	9.4[e]	9.7[l]	9.7[l]	0.6323
	(5.9-9.3)	(2.9)	(6.0-9.8)	(3.6)		(7.6-11.0)	(2.9)	(7.9-11.1)	(3.5)	
HAEMATOLOGY/COAGULATION										
white blood cell count, 10^9/l	12.7	14.81	12.69	13.91	0.3934	12.26	15.93	12.33	13.43	0.0272
	(9.78-18.19)	(8.63)	(9.69-16.02)	(7.75)		(9.65-18.18)	(10.80)	(9.46-15.71)	(6.28)	
international normalized ratio	1.12[b]	1.2[b]	1.075[a]	1.1[a]	0.1046	1.1[d]	1.2[d]	1.05[j]	1.1[j]	0.0001
	(1.02-1.24)	(0.32)	(1.01-1.16)	(0.22)		(1.02-1.22)	(0.2)	(0.99-1.1)	(0.1)	
aPTT, sec	27.1[b]	31.5[b]	25.45[c]	27.4[c]	0.0229	29.05[c]	33.2[c]	25.1[j]	28.4[j]	0.0052
	(23.1-33.2)	(16.0)	(23.15-28.80)	(11.1)		(24-38.05)	(14.9)	(22.7-30.1)	(10.6)	
red blood cell count, 10^{12}/l	3.4	3.49	3.38	3.44	0.5861	2.99	3.10	3.02	3.07	0.7162
	(2.91-3.97)	(0.77)	(2.95-3.94)	(0.69)		(2.75-3.35)	(0.54)	(2.71-3.39)	(0.50)	
hematocrit, %	30	30.89	29	30.28	0.4446	27	27.92	27	27.47	0.4494
	(26-35)	(6.48)	(26-34)	(6.00)		(25-30)	(4.93)	(25-30)	(4.31)	
MARKERS OF INFLAMMATION										
procalcitonin, μg/l	2.365[m]	14.8[m]	3.96[n]	15.5[n]	0.9031	1.755[o]	9.9[o]	1.13[s]	6.5[s]	0.4238
	(0.67-13.395)	(35.8)	(0.63-14.72)	(30.3)		(0.625-6.425)	(26.1)	(0.42-4.37)	(17.8)	
CLINICAL CHEMISTRY										
lactate, mmol/l	1.8	2.8	1.4	2.3	0.1651	1.4	2.4	1.1	1.3	$<$0.0001
	(1.1-3.3)	(2.9)	(0.9-2.4)	(2.6)		(1.0-2.6)	(2.5)	(0.8-1.5)	(1.0)	
calcium, mmol/l	1.18	1.2	1.19	1.2	0.2801	1.12	1.10	1.15	1.10	0.0385
	(1.13-1.23)	(0.1)	(1.15-1.24)	(0.1)		(1.05-1.19)	(0.13)	(1.10-1.18)	(0.10)	
potassium, mmol/l	4.2	4.2	4.1	4.2	0.9498	4.3	4.4	4.1	4.2	0.0011
	(3.8-4.6)	(0.7)	(3.8-4.6)	(0.8)		(4.0-4.8)	(0.6)	(3.8-4.4)	(0.4)	
urea, mg/dl	51.9	60.8	44.2	55.8	0.3195	79.7	89.0	56.0[a]	61.9[a]	$<$0.0001
	(31.7-85.7)	(39.1)	(30.0-73.6)	(38.9)		(45.1-128.9)	(55.4)	(36.9-79.4)	(33.3)	
alanine aminotransferase, U/l	26[g]	54.5[g]	23[f]	73.9[f]	0.4347	39[p]	137.4[p]	43[u]	75.9[u]	0.0922
	(17.0-51.5)	(104.2)	(17-50)	(246.8)		(22-92)	(250.8)	(25-72)	(101.3)	
aspartate aminotransferase, U/l	39[j]	101.6[j]	34.5[g]	120.7[g]	0.7095	57.5[o]	415.0[o]	47[u]	96.5[u]	0.0156
	(25-63)	(290.2)	(19-69)	(467.9)		(35.5-177.5)	(975.9)	(30-103)	(149.9)	
albumin, g/l	20.7[i]	20.7[i]	22[g]	21.9[g]	0.2204	15.0[r]	15.8[r]	17.45[v]	17.3[v]	0.2621
	(15.2-26.2)	(7.3)	(15.75-28.15)	(7.2)		(10.9-20.4)	(6.8)	(13.1-21.0)	(5.9)	
lipase, U/l	120[h]	264.2[h]	110[k]	268.4[k]	0.9525	124[u]	485.6[u]	130.5[w]	390.7[w]	0.6312
	(57-216)	(521.3)	(61-201)	(537.4)		(43-325)	(1066.0)	(72.5-327.0)	(665.2)	

S2.2 Scatterplots and Spearman correlations

For the four considered networks in our analysis of the 123 matched case-control pairs, pairwise scatterplots of the corresponding underlying network parameter data, along with pairwise Spearman correlation coefficients, are given in Figures S1 (non-survivors at admission), S2 (survivors at admission), S3 (non-survivors at event) and S4 (survivors at event).
Overall, the Spearman correlations are rather weak to moderate throughout (with a maximum absolute value of 0.54 for the association between bilirubin and platelet counts in the survivor event network).
For the non-survivor group, absolute Spearman correlations are typically weaker at admission stage (average absolute correlation: 0.12) and remain weak or only get marginally stronger at event stage (average absolute correlation: 0.13). In contrast, for the survivor group, absolute Spearman correlations are typically weaker at admission stage (average absolute correlation: 0.15) and get stronger at event stage (average absolute correlation: 0.19).
Already in view of the additional subgroup analysis performed in Supplement S3, comprising only encounters not receiving renal replacement therapy (RRT; dialysis) during ICU stay, orange points in the scatterplots indicate encounters that do receive RRT at the time point of interest (non-survivors admission: 12 encounters; non-survivors event: 30; survivors admission: 9; survivors event: 26). There are clearly more encounters with RRT at the event stage than at the admission stage. At admission, encounters with RRT often show higher levels of creatinine. At event stage, encounters receiving RRT typically have lower values of sodium in both the non-survivor and the survivor group, and lower levels of platelet counts as well as higher values of bilirubin in the non-survivor group.

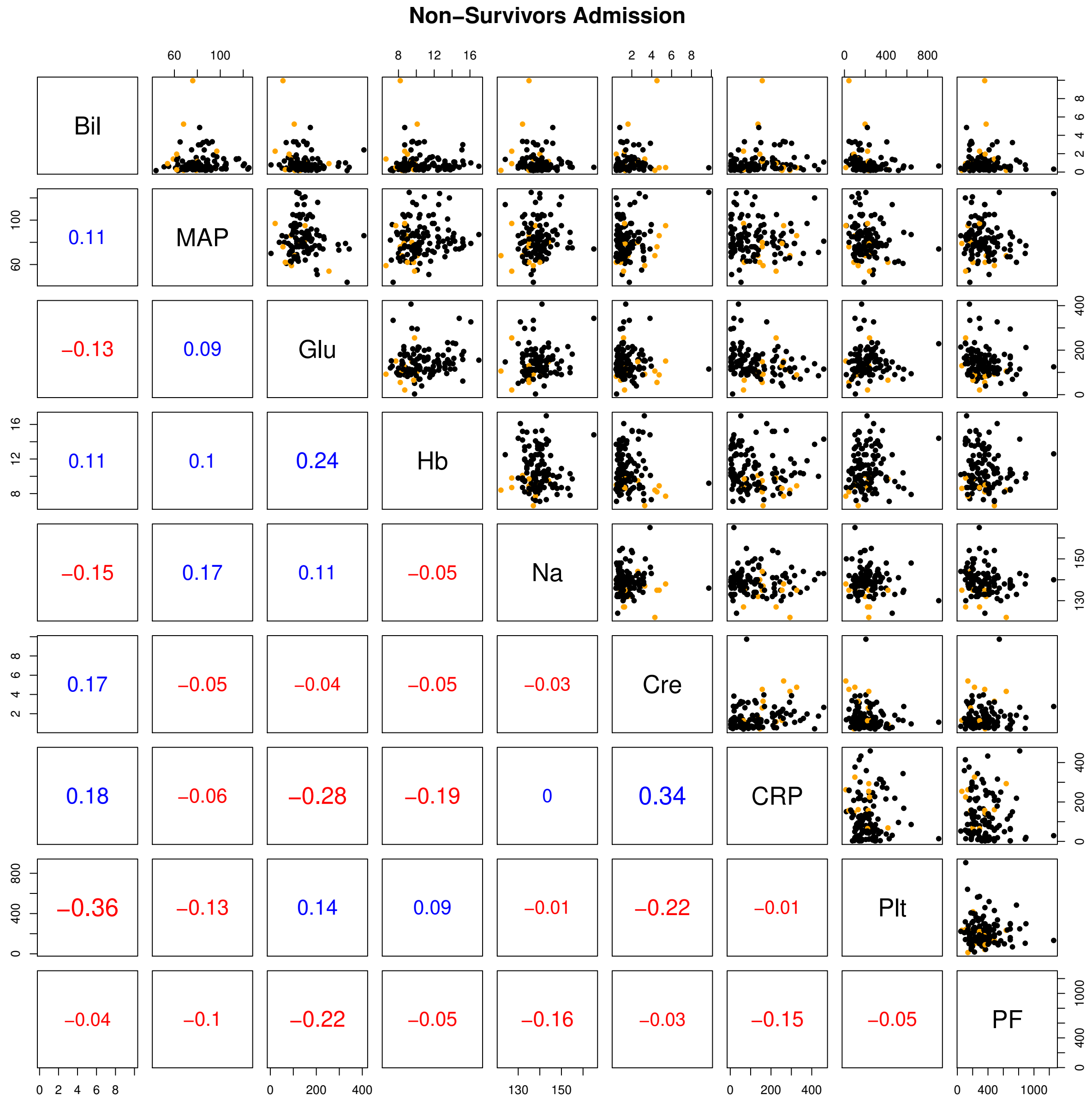

Figure S1: Pairwise scatterplots and corresponding Spearman correlations for the network parameters for the non-survivor group at admission stage. Orange points indicate encounters receiving RRT.

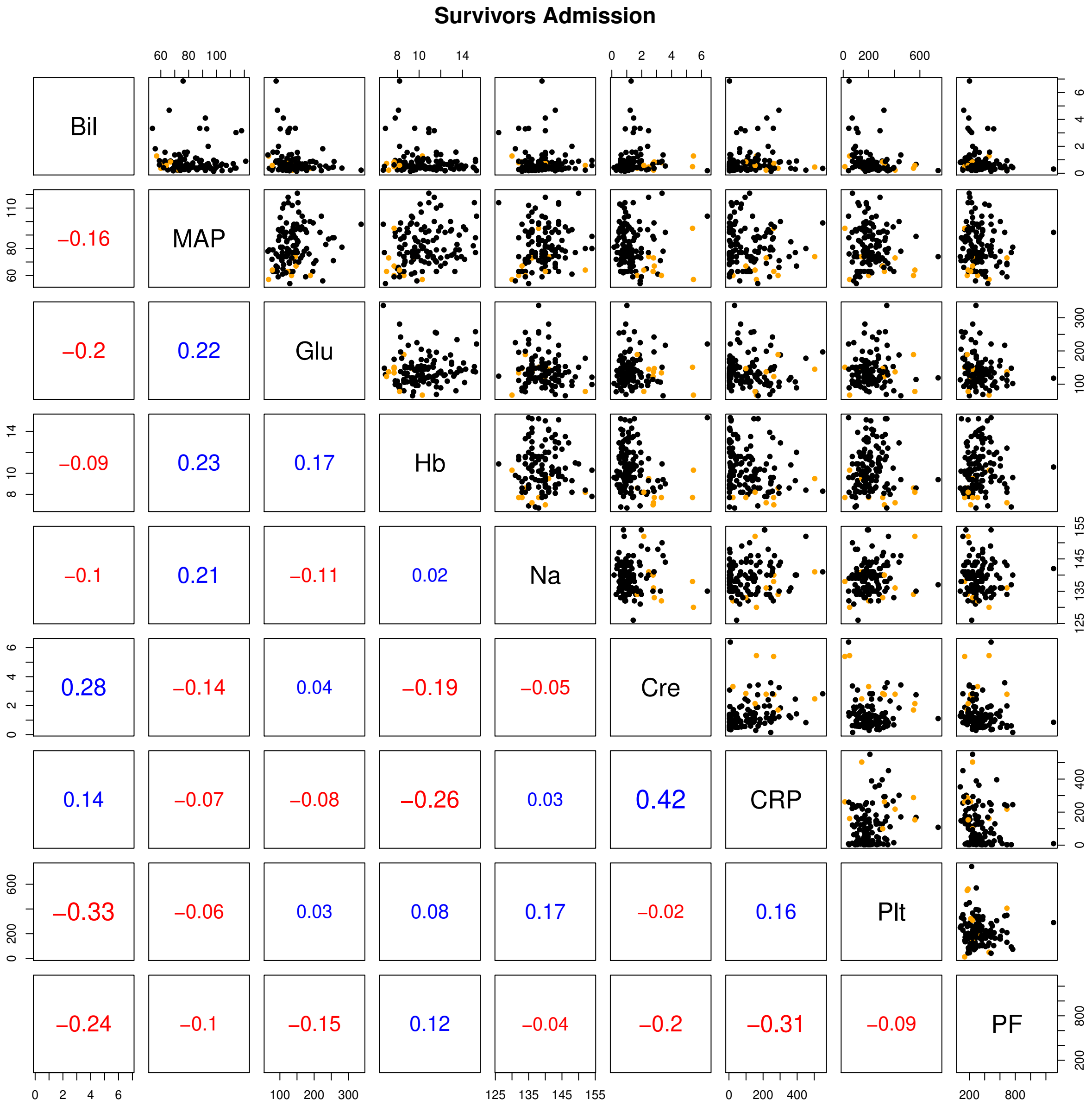

Figure S2: Pairwise scatterplots and corresponding Spearman correlations for the network parameters for the survivor group at admission stage. Orange points indicate encounters receiving RRT.

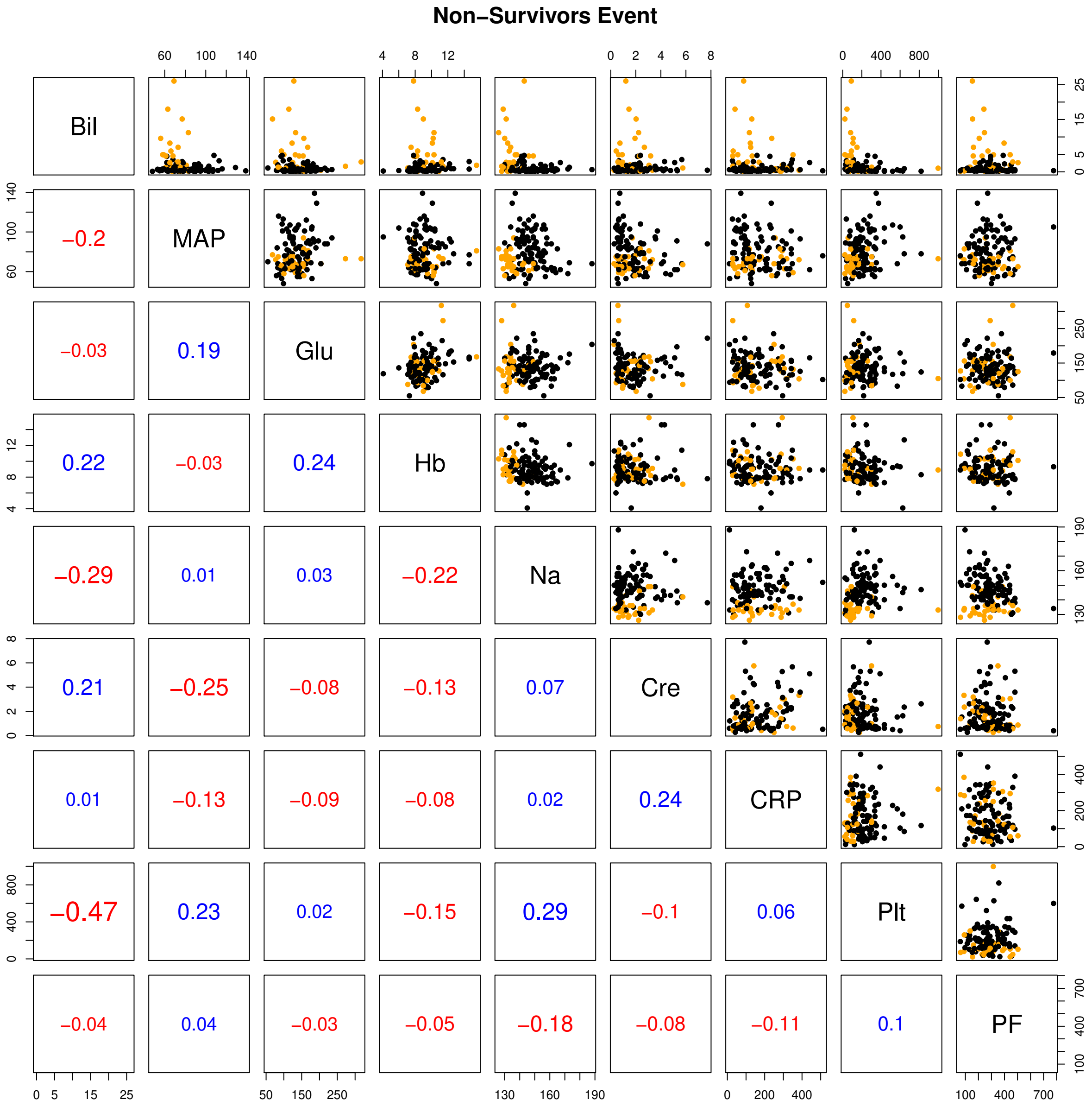

Figure S3: Pairwise scatterplots and corresponding Spearman correlations for the network parameters for the non-survivor group at event stage. Orange points indicate encounters receiving RRT.

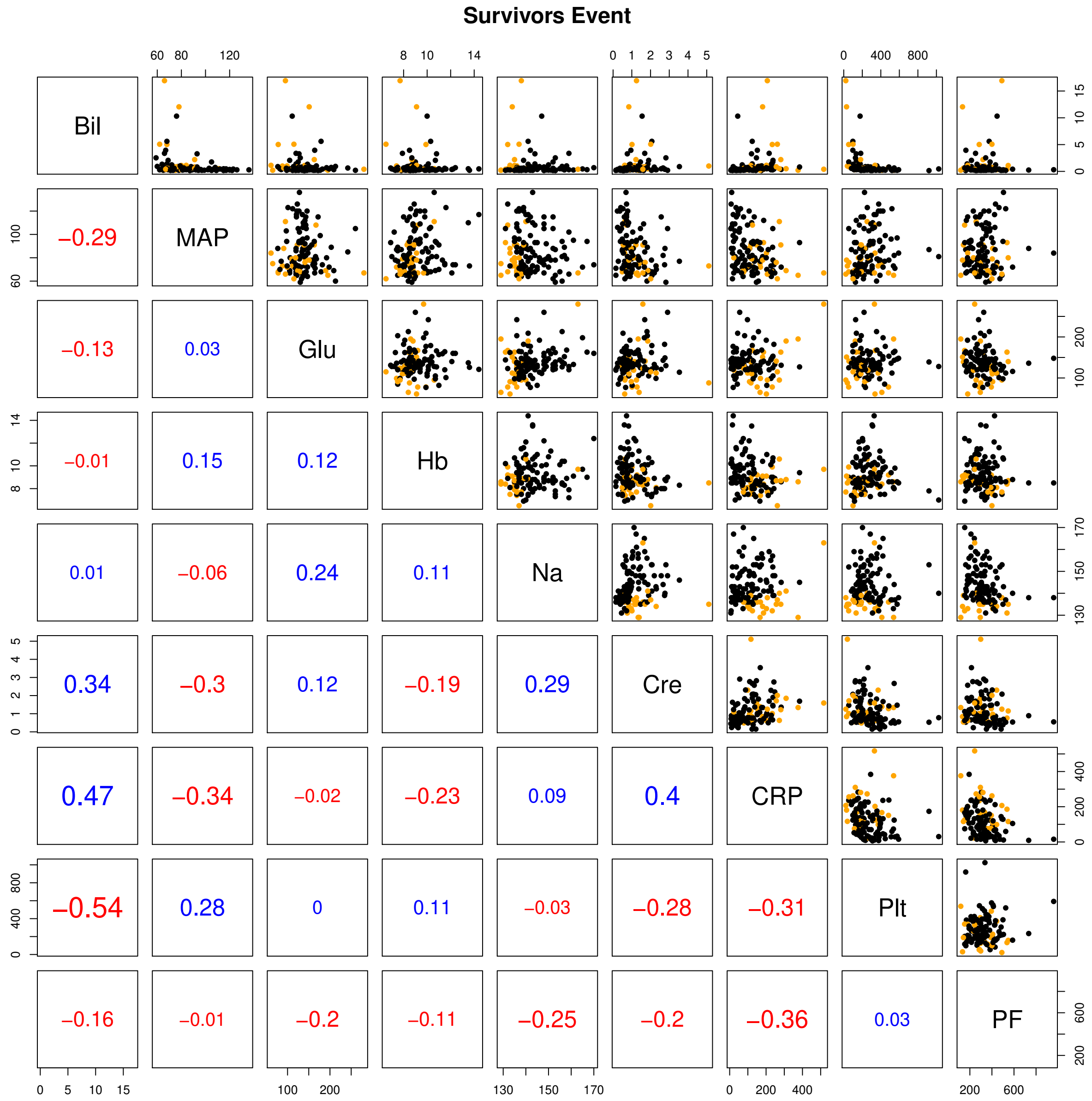

Figure S4: Pairwise scatterplots and corresponding Spearman correlations for the network parameters for the survivor group at event stage. Orange points indicate encounters receiving RRT.

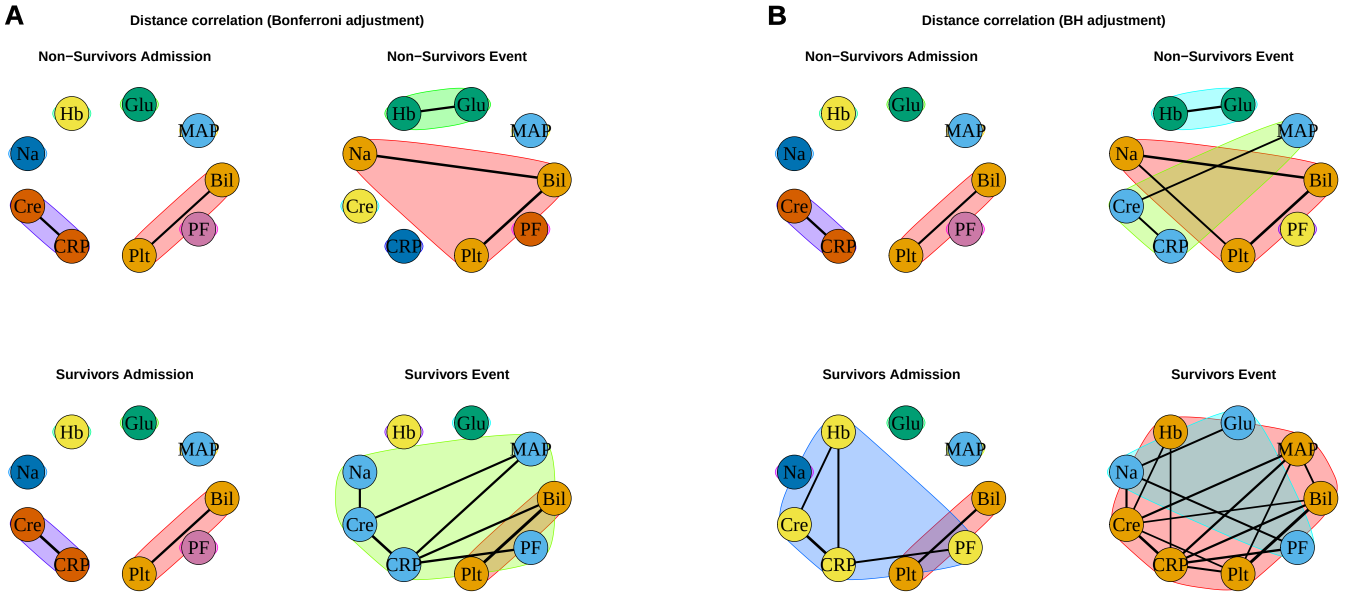

Figure S5: Networks estimated based on distance correlations together with (A) Bonferroni and (B) BH adjustment, respectively. The thickness of the edges refers to the absolute magnitude of the correlation (the higher the correlation the thicker the edge).

S2.3 Results for distance correlation

Here, we re-perform the analyses from the main text, using network estimation based on distance correlation (instead of Spearman correlations), together with Bonferroni and BH adjustment, respectively (Figure S5; Tables S2 and S3). The distance correlation can measure both linear and non-linear association between two variables. It can attain non-negative values in $[0, 1]$, not values in $[-1, 1]$ as the Spearman correlation. Moreover, the distance correlation between two variables is zero if and only if the two variables are independent, and it is one in case of a perfect linear dependence.

To determine whether a distance correlation value is statistically significant (and thus whether a corresponding edge is drawn in the network), the corresponding p-value is derived using another permutation test (apart from the permutation test to check for differences between two networks). Due to clearly increased computational running time in this setting, for our purposes here, we limit our analysis to the use of $M = 100$ permutations only to compute a p-value to check for differences between two networks.

Essentially, the networks overall look fairly similar to those estimated by Spearman correlations (main text), and basically, the results of our original analysis are confirmed.

In particular, the diverging dynamics between survivors and non-survivors in the course of the ICU stay can be observed and confirmed (no significant overall network differences for comparisons (C1) and (C4), significant differences with respect to global strength, Frobenius metric, Jaccard distance and number of edges for comparison (C2), significant differences in terms of global strength, Frobenius metric, spectral distance and number of edges for comparison (C3); Tables S2 and S3).

On the node-specific level, when comparing the networks for non-survivors and survivors at the event stage (C2), C-reactive protein (CRP) is confirmed to show a significantly different degree, with no or one edge(s) in the non-survivor event network and many edges in the survivor event network (Figure S5; $P < 0.01$, Tables S2 and S3). Similarly, regarding the longitudinal comparison between admission and event stage for the survivor group (C3), sodium ($P < 0.01$, Table S3) and platelet counts ($P < 0.01$, Table S3) are confirmed to reveal a significantly different degree with many

edges at the event stage and none at admission (Figure S5B).
On the edge-specific level, for comparison (C2), the association between bilirubin and CRP ($P < 0.01$, Tables S2 and S3) is confirmed to be significantly different, in that it is present in the survivor group, but not in the non-survivor group at the event stage. Regarding comparison (C3), the associations between bilirubin and CRP, between sodium and creatinine and between CRP and platelet counts are confirmed to be present at the event stage, but not at the admission stage for the survivor group (Tables S2 and S3).
Finally, as for the Spearman correlation-based networks, the association between bilirubin and platelet counts forms the only edge that is present in all networks.

Table S2: P-values corresponding to the cross-sectional and longitudinal comparisons between networks estimated using distance correlations together with Bonferroni adjustment (Figure S5A) for different network difference characteristics: global strength, Frobenius metric, maximum metric, spectral distance, Jaccard distance, number of edges, number of clusters, number of isolated nodes, degree of a specific node i (only nodes corresponding to a p-value $\leq$ 0.05 are shown), edge strength between two specific nodes i and j (only edges corresponding to a p-value $\leq$ 0.05 are shown). P-values $\leq$ 0.05 are indicated in bold font.

		cross-sectional		longitudinal	
		(C1) non-survivors admission vs. survivors admission	(C2) non-survivors event vs. survivors event	(C3) survivors admission vs. survivors event	(C4) non-survivors admission vs. non-survivors event
overall	global strength	0.84	0.26	**0.01**	0.49
	Frobenius metric	0.97	**0.02**	0.14	0.40
	maximum metric	0.97	0.56	0.77	0.58
	spectral distance	0.91	0.47	0.10	0.75
	Jaccard distance	1.00	**0.03**	0.21	0.56
	number of edges	1.00	0.28	**0.01**	0.81
	number of clusters	1.00	0.37	0.06	0.78
	number of isolated nodes	1.00	0.53	0.13	0.81
nodes	degree of node i	none	CRP: <**0.01**	none	none
edges	edge strength between nodes i and j	none	Bil-CRP: <**0.01**<br>Bil-Na: <**0.01**<br>MAP-CRP: **0.04**	Bil-CRP: <**0.01**<br>Na-Cre: <**0.01**	Bil-Na: <**0.01**

Table S3: P-values corresponding to the cross-sectional and longitudinal comparisons between networks estimated using distance correlations together with BH adjustment (Figure S5B) for different network difference characteristics: global strength, Frobenius metric, maximum metric, spectral distance, Jaccard distance, number of edges, number of clusters, number of isolated nodes, degree of a specific node i (only nodes corresponding to a p-value ≤ 0.05 are shown), edge strength between two specific nodes i and j (only edges corresponding to a p-value ≤ 0.05 are shown). P-values ≤ 0.05 are indicated in bold font.

		cross-sectional		longitudinal	
		(C1)<br>non-survivors admission<br>vs.<br>survivors admission	(C2)<br>non-survivors event<br>vs.<br>survivors event	(C3)<br>survivors admission<br>vs.<br>survivors event	(C4)<br>non-survivors admission<br>vs.<br>non-survivors event
overall	global strength	0.43	**0.01**	**<0.01**	0.26
	Frobenius metric	0.85	**<0.01**	**<0.01**	0.63
	maximum metric	0.93	0.43	0.56	0.41
	spectral distance	0.52	0.15	**<0.01**	0.24
	Jaccard distance	0.88	**0.02**	0.38	0.72
	number of edges	0.51	**0.04**	**<0.01**	0.29
	number of clusters	0.55	0.44	0.11	0.28
	number of isolated nodes	0.69	0.86	0.15	0.26
nodes	degree of node i	none	CRP: **<0.01**	Na: **<0.01**<br>Plt: **<0.01**<br>Cre: **0.03**<br>MAP: **0.05**	none
edges	edge strength between nodes i and j	none	Bil-CRP: **<0.01**<br>Bil-Na: **<0.01**<br>Glu-Na: **<0.01**<br>CRP-Plt: **<0.01**<br>Na-Plt: **0.02**	Bil-CRP: **<0.01**<br>Glu-Na: **<0.01**<br>Na-Cre: **<0.01**<br>CRP-Plt: **<0.01**<br>Na-PF: **0.02**<br>MAP-Plt: **0.03**<br>MAP-CRP: **0.04**	Bil-Na: **<0.01**<br>Na-Plt: **0.01**

S2.4 Results for EBICglasso

Here, we re-perform the analyses from the main text, using network estimation based on the EBICglasso approach (instead of Spearman correlations), which combines network estimation based on partial correlation with lasso regularization and model selection. In particular, lasso regularization is conducted when estimating a network via partial correlations, and by varying the lasso tuning parameter λ, a collection of possible networks is obtained. From this collection, a final "best" network is selected by choosing a model based on the minimization of the extended Bayesian information criterion (EBIC), which in turn depends on a hyperparameter γ, typically $\gamma \in [0, 0.5]$, controlling how much the EBIC prefers simpler models.

In our considerations here, we focus on the EBICglasso method with a tuning parameter of $\gamma = 0.25$ (Figure S6 and Table S4). This choice of γ appears to be a reasonable trade-off between being too conservative ($\gamma \to 0.5$) and including too many spurious edges ($\gamma \to 0$) for our purposes here. To underline this, for visual inspection only, we have added the networks obtained via the EBICglasso approach using varying tuning parameters $\gamma \in \{0, 0.1, 0.2, 0.3, 0.4, 0.5\}$ (Figure S7). It can be confirmed that smaller values of γ ($\gamma \in \{0, 0.1\}$) lead to the inclusion of more (possibly spurious) edges into the network. With increasing values of γ, more and more edges vanish, and from values of $\gamma = 0.2$ on, edges at the admission stage even disappear drastically. From values of $\gamma = 0.4$ on, only quite few edges actually remain, which makes it difficult to see any differences at all, where however, most edges appear in the survivor event network.

Based on the above observations, we believe that $\gamma = 0.25$ is indeed a reasonable choice in our setting. Overall, for EBICglasso with $\gamma = 0.25$, the networks may look a bit different (Figure S6) than those in our original analysis based on Spearman correlations, with fewer edges drawn (e.g., no edges at all for non-survivor admission and survivor admission networks), which can be expected due to the more conservative concept of partial correlations that is involved. However, the survivor event network also has by far the most edges, and the differential network testing results obtained before are basically confirmed.

In particular, the diverging dynamics between survivors and non-survivors in the course of the ICU stay can be observed and confirmed (no significant overall network differences for comparisons (C1) and (C4), significant differences with respect to global strength and number of edges for comparison (C2), significant differences in terms of all overall network difference characteristics for comparison (C3); Table S4).

On the node-specific level, when comparing the networks for non-survivors and survivors at the event stage (C2), CRP is confirmed to show a significantly different degree, with no edge in the non-survivor event network and many edges in the survivor event network (Figure S6; $P = 0.0157$, Table S4). Similarly, regarding the longitudinal comparison between admission and event stage for the survivor group (C3), sodium ($P = 0.0062$, Table S4) and platelet counts ($P = 0.0001$, Table S4) are confirmed to reveal a significantly different degree with many edges at the event stage and none at admission (Figure S6).

On the edge-specific level, for comparison (C2), the association between bilirubin and CRP ($P = 0.0003$, Table S4) is confirmed to be significantly different, in that it is present in the survivor group, but not in the non-survivor group at the event stage. Regarding comparison (C3), the positive association between bilirubin and CRP ($P < 0.0001$), the positive association between sodium and creatinine ($P = 0.0006$) and the negative association between CRP and platelet counts ($P = 0.0012$) are confirmed to be present at the event stage, but not at the admission stage for the survivor group (Table S4).

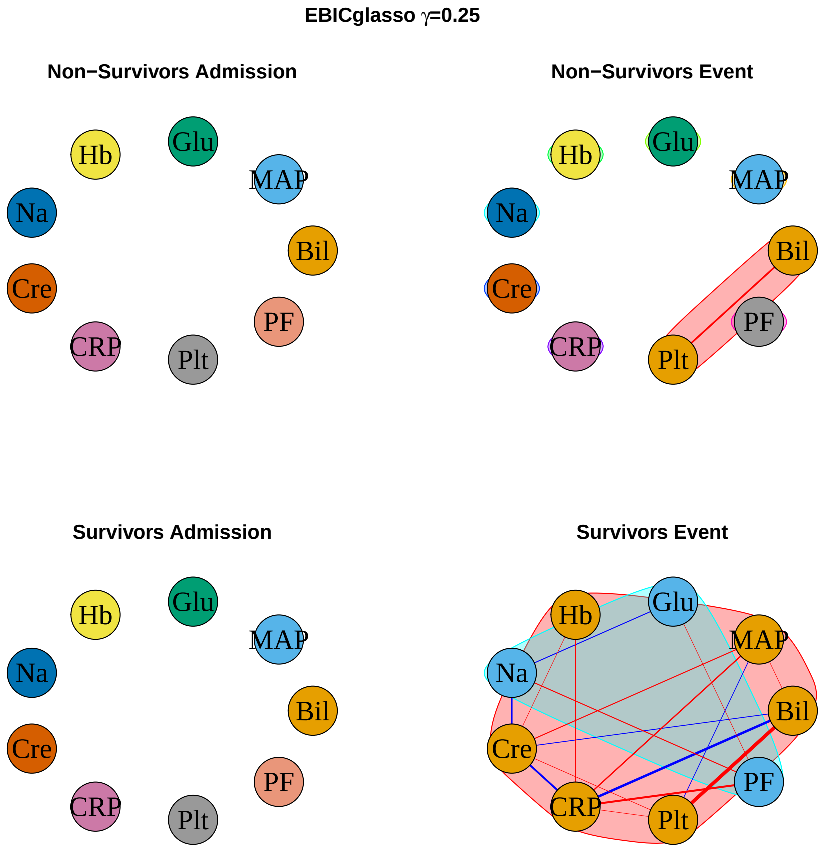

Figure S6: Networks estimated based on EBICglasso approach with tuning parameter $\gamma = 0.25$. Positive associations are indicated in blue, and negative associations in red. The thickness of the edges refers to the absolute magnitude of the correlation (the higher the correlation the thicker the edge).

Table S4: P-values corresponding to the cross-sectional and longitudinal comparisons between networks estimated using EBICglasso with tuning parameter $\gamma = 0.25$ (Figure S6) for different network difference characteristics: global strength, Frobenius metric, maximum metric, spectral distance, Jaccard distance, number of edges, number of clusters, number of isolated nodes, degree of a specific node i (only nodes corresponding to a p-value ≤ 0.05 are shown), edge strength between two specific nodes i and j (only edges corresponding to a p-value ≤ 0.05 are shown). P-values ≤ 0.05 are indicated in bold font.

		cross-sectional		longitudinal	
		(C1) non-survivors admission vs. survivors admission	(C2) non-survivors event vs. survivors event	(C3) survivors admission vs. survivors event	(C4) non-survivors admission vs. non-survivors event
overall	global strength	1.0000	**0.0423**	**0.0031**	0.5378
	Frobenius metric	1.0000	0.0703	**0.0099**	0.4877
	maximum metric	1.0000	0.1489	**0.0348**	0.3712
	spectral distance	1.0000	0.0703	**0.0090**	0.4371
	Jaccard distance	1.0000	0.0593	0.1162	0.4364
	number of edges	1.0000	**0.0329**	**0.0012**	0.8164
	number of clusters	1.0000	0.0589	**0.0137**	0.8135
	number of isolated nodes	1.0000	0.1152	**0.0153**	0.7990
nodes	degree of node i	none	CRP: **0.0157**	Plt: **0.0001**<br>Cre: **0.0010**<br>CRP: **0.0016**<br>Na: **0.0062**	none
edges	edge strength between nodes i and j	none	Bil-CRP: **0.0003**<br>Glu-Na: **0.0173**<br>CRP-PF: **0.0267**<br>Glu-PF: **0.0407**<br>Na-Cre: **0.0423**<br>Hb-CRP: **0.0478**	Bil-CRP: **<0.0001**<br>Na-Cre: **0.0006**<br>CRP-Plt: **0.0012**<br>Glu-Na: **0.0014**<br>MAP-CRP: **0.0132**<br>Cre-Plt: **0.0148**<br>MAP-Plt: **0.0152**<br>Na-PF: **0.0175**<br>Bil-Plt: **0.0339**	none

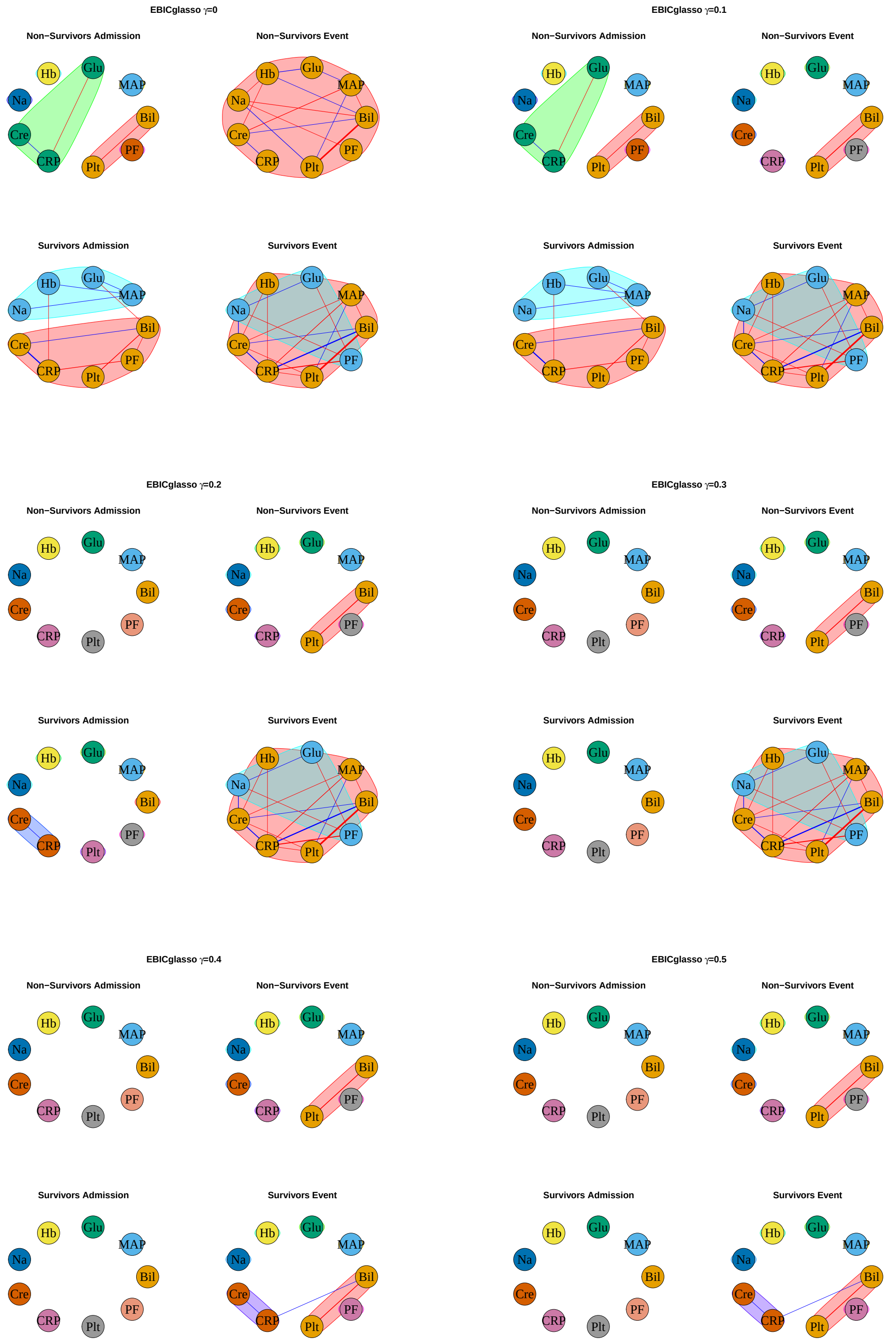

Figure S7: Networks estimated based on EBICglasso approach with tuning parameters $\gamma \in \{0, 0.1, 0.2, 0.3, 0.4, 0.5\}$. Positive associations are indicated in blue, and negative associations in red. The thickness of the edges refers to the absolute magnitude of the correlation (the higher the correlation the thicker the edge).

S3 Supplementary results: Subgroup analyses for encounters not receiving renal replacement therapy (RRT; dialysis)

To investigate the effect of medical intervention on our results, we here exemplarily consider renal replacement therapy (RRT; dialysis) as one of the most frequent therapies and additionally re-perform our analyses from the main text for the subgroup of encounters that do not receive RRT at admission or event stage.
In total, referring to the 123 original matched case-control pairs, there are 27 encounters in the survivor group receiving RRT either at admission or at event stage, and 31 encounters in the non-survivor group receiving RRT either at admission or at event stage. Moreover, we observe 77 matching pairs without RRT at either admission or event stage for either cases (non-survivors) or controls (survivors), and consequently 46 matching pairs receiving RRT at either admission or event stage for either cases (non-survivors) or controls (survivors).
In what follows, for the 77 matching pairs without any RRT, we perform the same analyses as in the main text, thus with a focus on Spearman correlations, together with Bonferroni and BH adjustment, respectively.
First, for the four considered networks, we show pairwise scatterplots of the corresponding underlying network parameter data, along with pairwise Spearman correlation coefficients, in Figures S8 (non-survivors at admission), S9 (survivors at admission), S10 (non-survivors at event) and S11 (survivors at event). Overall, the Spearman correlations are rather weak to moderate throughout (with a maximum absolute value of 0.51 for the association between bilirubin and platelet counts in the survivor event network). For the non-survivor group, absolute Spearman correlations are typically weaker at admission stage (average absolute correlation: 0.15) and remain weak or only get marginally stronger at event stage (average absolute correlation: 0.16). In contrast, for the survivor group, absolute Spearman correlations are typically weaker at admission stage (average absolute correlation: 0.12) and get stronger at event stage (average absolute correlation: 0.23). Thus, we essentially observe similar data structure and tendencies for our subgroup here than for the original data set.
Moreover, when considering Spearman correlation-based networks, basically very similar results hold for the subgroup as for the original data including all matching pairs (main text), see Figure S12 and Tables S5 and S6.
In particular, similar diverging network dynamics for survivors and non-survivors during ICU stay can be observed (no significant overall network differences for the comparisons (C1) and (C4), significant differences with respect to global strength, spectral distance, Jaccard distance, number of edges, number of clusters and number of isolated nodes for comparison (C2), significant differences in terms of global strength, Frobenius metric, spectral distance and number of edges for comparison (C3); Tables S5 and S6).
On the node-specific level, when comparing the networks for non-survivors and survivors at the event stage (C2), CRP reveals a significantly different degree, with no edge in the non-survivor event network and many edges in the survivor event network (Figure S12; $P = 0.0136$, Table S5, and $P = 0.0261$, Table S6). Similarly, regarding the longitudinal comparison between admission and event stage for the survivor group (C3), sodium shows a significantly different degree with many edges at the event stage and none at admission (Figure S12; $P < 0.0001$, Table S5, and $P = 0.0016$, Table S6).
On the edge-specific level, regarding comparison (C3), the positive association between bilirubin and CRP ($P = 0.0002$, Tables S5 and S6), the positive association between sodium and creatinine ($P = 0.0019$, Tables S5 and S6) and the negative association between CRP and platelet counts ($P = 0.0005$, Tables S5 and S6) are present at the event stage, but not at the admission stage for the survivor group.

In what follows, we compare the results of the above subgroup analysis to the results obtained for the overall patient group (main text).
In summary, with respect to the overall network difference characteristics, the results of the overall analyses for all encounters basically continue to hold for the subgroup of encounters not receiving RRT, thus showing that an intervention by RRT has only a minor influence on the analyses of overall network structures, if any.
However, partly different results are obtained with respect to the node- and edge-specific network differences characteristics, respectively.
Regarding differences in node degrees, the results are still similar for the comparisons (C1) and (C2). For the compari-

son (C3), this also holds for the Bonferroni adjustment, where for the BH adjustment, more nodes show a significantly different degree for the subgroup of encounters not receiving RRT than for the overall patient group, with both groups revealing sodium and platelet counts as parameters with significantly different degrees. For the comparison (C4), the significantly different degree for platelet counts (and mean arterial pressure, MAP) that is found in the overall patient group vanishes when considering the subgroup of encounters not receiving RRT.
In terms of differences in edge strength, on the one hand, the results are still similar for the comparisons (C1) and (C3). On the other hand, for the comparisons (C2) and (C4), all edges that have been found to be significantly different for the overall patient group are not significantly different anymore for the subgroup of encounters not receiving RRT. Most prominently, for the overall patient group, a positive association between sodium and platelet counts is only present in the non-survivor event network, yielding significant differences with respect to edge strength for the comparisons (C2) ($P = 0.0108$, Tables 3 and 4 in the main text) and (C4) ($P = 0.0086$, Tables 3 and 4 in the main text). In the subgroup of patients not receiving RRT, this sodium-platelet counts association vanishes, and so do the respective significant differences for (C2) and (C4) (Figure S12; Tables S5 and S6). This can be explained as follows. For non-survivors at the event stage, patients receiving an RRT are typically associated with lower values of sodium and a lower number of platelet counts, see (the orange points in) the corresponding scatterplot for the sodium-platelet counts association in Figure S3. This makes sense from a clinical perspective, as RRT is known to reduce platelet counts [1] and to remove sodium [2]. This block of encounters is not present in the subgroup of patients not receiving RRT any longer, see the corresponding scatterplot for the sodium-platelet counts association in Figure S10. This weakens the statistically significant positive association between sodium and platelet counts of 0.29 in the overall patient group to a non-significant positive association of 0.19 in the subgroup of patients not receiving RRT. Thus, the sodium-platelet association is not present in any of the four considered networks for the subgroup of patients not receiving RRT, and consequently, the significant differences for comparison (C2) and (C4), which are observed in the overall patient group, vanish.

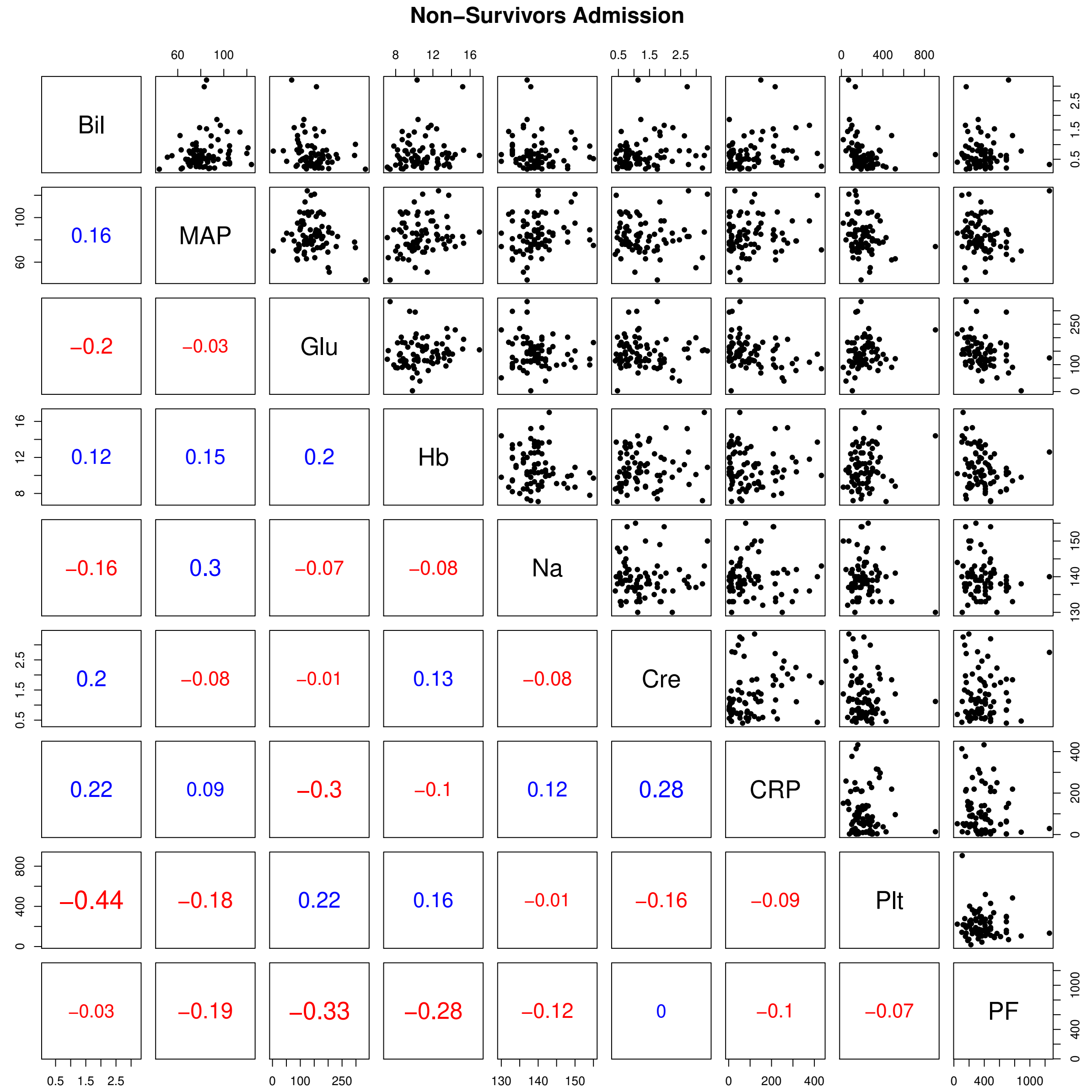

Figure S8: Pairwise scatterplots and corresponding Spearman correlations for the network parameters for the non-survivor group at admission stage, only including encounters not receiving RRT

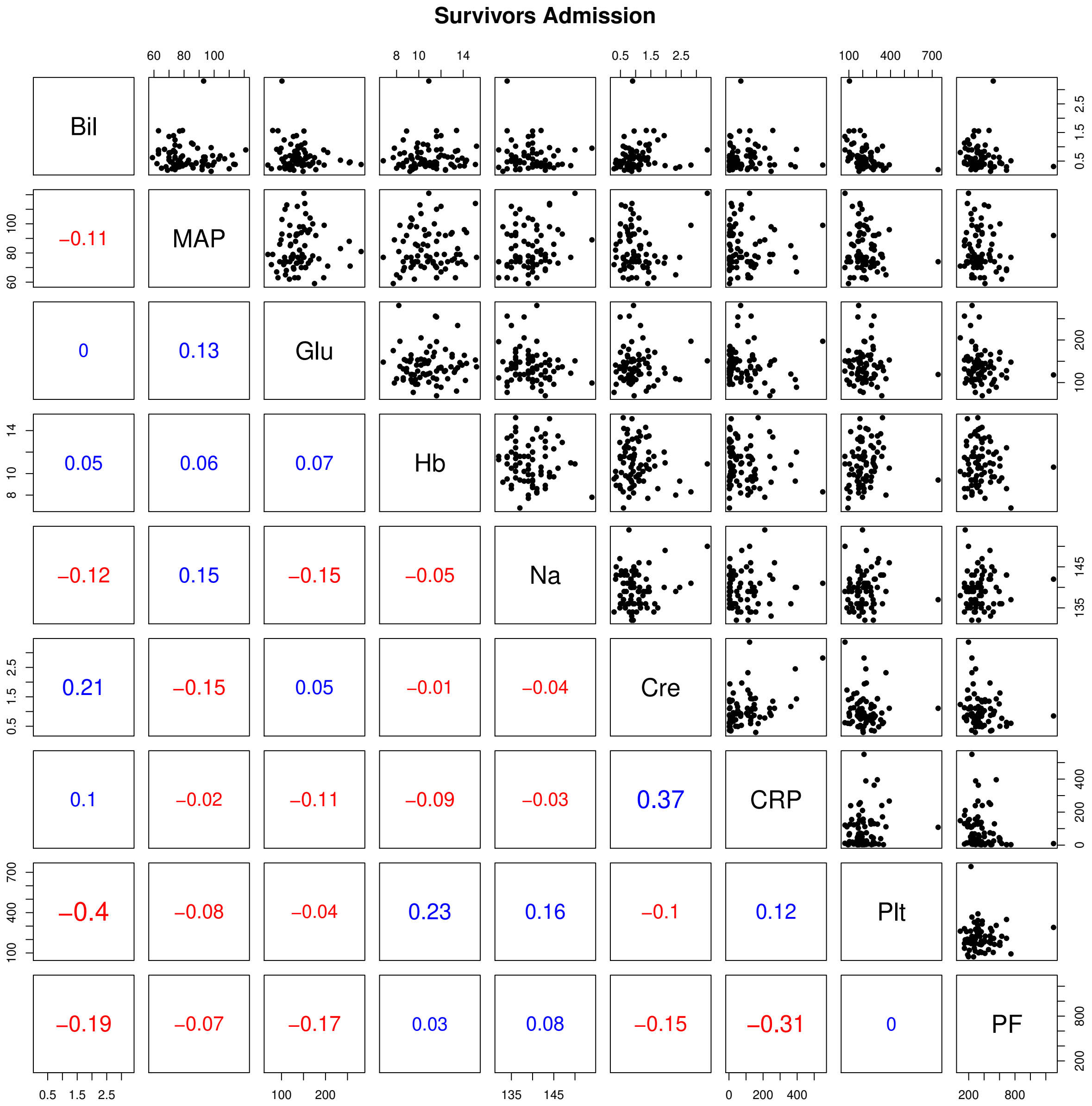

Figure S9: Pairwise scatterplots and corresponding Spearman correlations for the network parameters for the survivor group at admission stage, only including encounters not receiving RRT

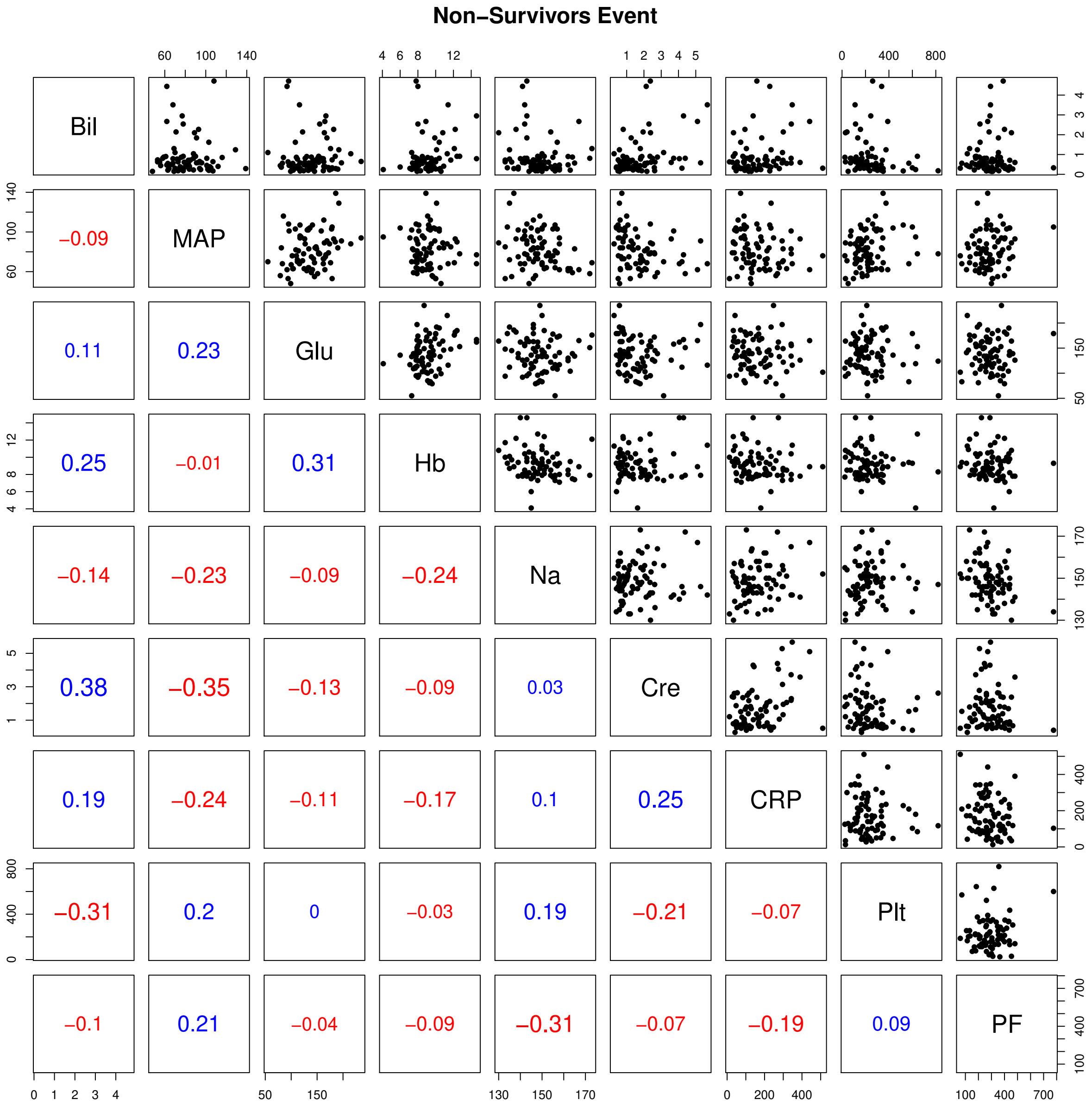

Figure S10: Pairwise scatterplots and corresponding Spearman correlations for the network parameters for the non-survivor group at event stage, only including encounters not receiving RRT

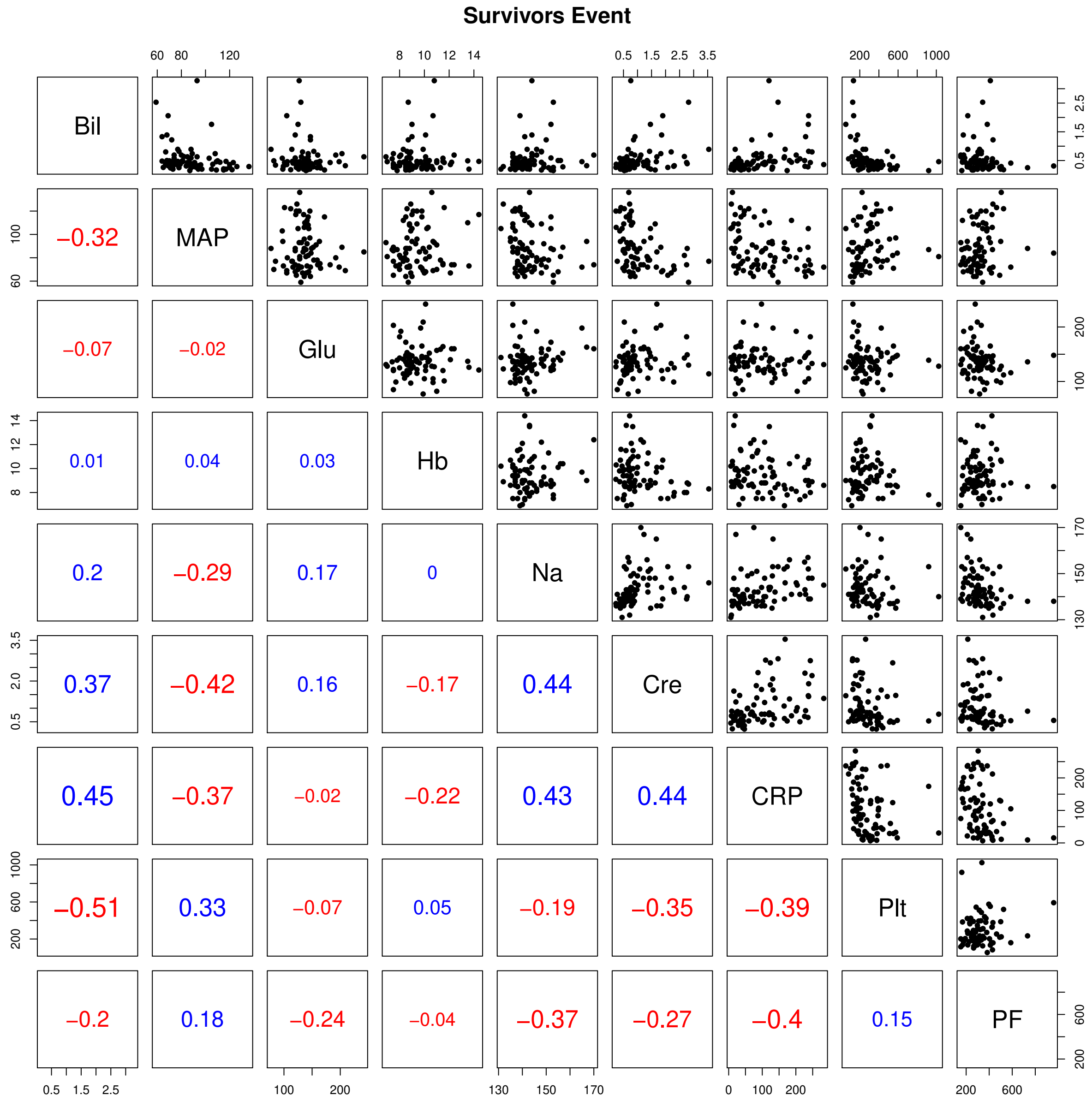

Figure S11: Pairwise scatterplots and corresponding Spearman correlations for the network parameters for the survivor group at event stage, only including encounters not receiving RRT

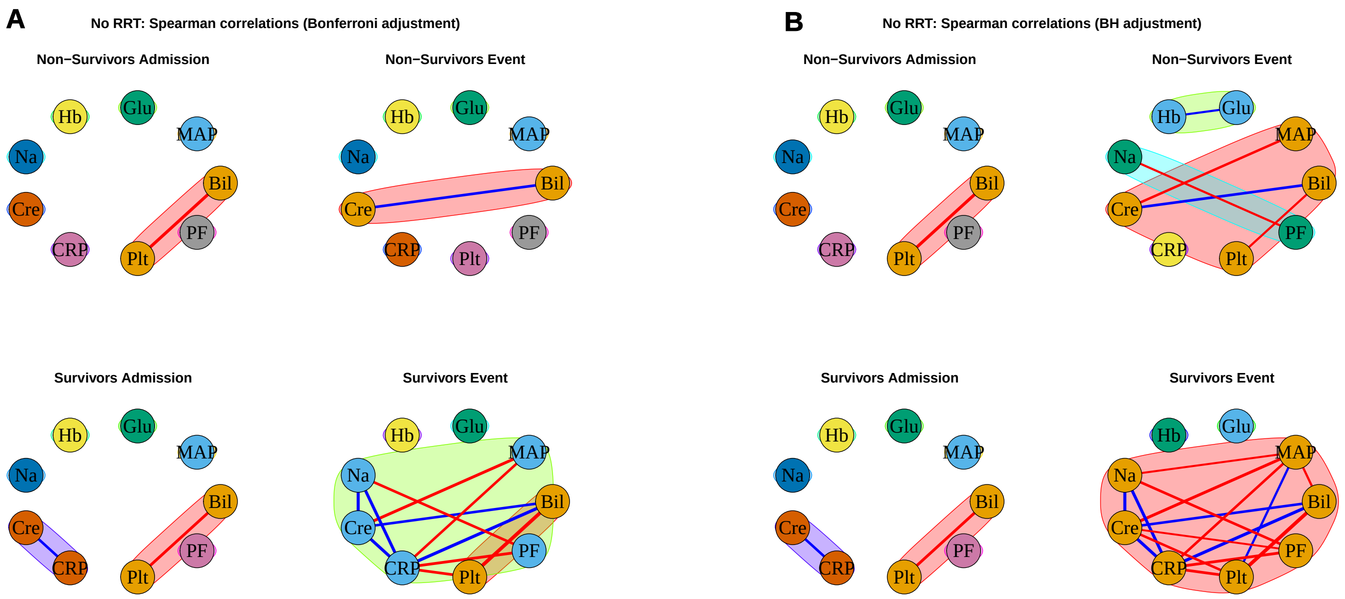

Figure S12: Networks estimated based on Spearman correlations together with (A) Bonferroni and (B) BH adjustment, respectively, for encounters not receiving RRT. Positive associations are indicated in blue, and negative associations in red. The thickness of the edges refers to the absolute magnitude of the correlation (the higher the correlation the thicker the edge).

Table S5: P-values corresponding to the cross-sectional and longitudinal comparisons for networks estimated using Spearman correlations together with Bonferroni adjustment for the subgroup of encounters not receiving RRT (Figure S12A) for different network difference characteristics: global strength, Frobenius metric, maximum metric, spectral distance, Jaccard distance, number of edges, number of clusters, number of isolated nodes, degree of a specific node i (only nodes corresponding to a p-value ≤ 0.05 are shown), edge strength between two specific nodes i and j (only edges corresponding to a p-value ≤ 0.05 are shown). P-values ≤ 0.05 are indicated in bold font.

		cross-sectional		longitudinal	
		(C1) non-survivors admission vs. survivors admission	(C2) non-survivors event vs. survivors event	(C3) survivors admission vs. survivors event	(C4) non-survivors admission vs. non-survivors event
overall	global strength	0.6519	**0.0222**	**<0.0001**	0.8837
	Frobenius metric	0.9178	0.1943	**<0.0001**	0.9043
	maximum metric	0.8951	0.4826	0.4219	0.6371
	spectral distance	0.7025	**0.0180**	**0.0009**	0.9193
	Jaccard distance	0.9422	0.1240	0.0642	0.6366
	number of edges	0.7582	**0.0225**	**<0.0001**	1.0000
	number of clusters	0.7529	**0.0359**	0.0515	1.0000
	number of isolated nodes	0.7199	**0.0260**	0.2559	1.0000
nodes	degree of node i	none	CRP: **0.0136**	CRP: **<0.0001**<br>Na: **<0.0001**<br>Bil: **0.0194**<br>PF: **0.0355**<br>Cre: **0.0418**	none
edges	edge strength between nodes i and j	none	none	Bil-CRP: **0.0002**<br>CRP-Plt: **0.0005**<br>Na-Cre: **0.0019**<br>Na-PF: **0.0043**<br>MAP-CRP: **0.0113**<br>Na-CRP: **0.0136**	none

Table S6: P-values corresponding to the cross-sectional and longitudinal comparisons for networks estimated using Spearman correlations together with BH adjustment for the subgroup of encounters not receiving RRT (Figure S12B) for different network difference characteristics: global strength, Frobenius metric, maximum metric, spectral distance, Jaccard distance, number of edges, number of clusters, number of isolated nodes, degree of a specific node i (only nodes corresponding to a p-value ≤ 0.05 are shown), edge strength between two specific nodes i and j (only edges corresponding to a p-value ≤ 0.05 are shown). P-values ≤ 0.05 are indicated in bold font.

		cross-sectional		longitudinal	
		(C1) non-survivors admission vs. survivors admission	(C2) non-survivors event vs. survivors event	(C3) survivors admission vs. survivors event	(C4) non-survivors admission vs. non-survivors event
overall	global strength	0.7153	0.0524	**<0.0001**	0.4090
	Frobenius metric	0.9461	0.1203	**<0.0001**	0.9544
	maximum metric	0.8505	0.6465	0.3684	0.9001
	spectral distance	0.6489	**0.0334**	**0.0004**	0.3042
	Jaccard distance	0.9447	**0.0499**	0.0677	0.6459
	number of edges	0.8440	0.0874	**<0.0001**	0.4061
	number of clusters	0.8245	0.8017	0.0631	0.2034
	number of isolated nodes	0.7455	0.7645	0.2794	0.1160
nodes	degree of node i	none	CRP: **0.0261**	MAP: **0.0014**<br>Na: **0.0016**<br>Plt: **0.0040**<br>CRP: **0.0056**<br>Cre: **0.0314**<br>PF: **0.0343**	none
edges	edge strength between nodes i and j	none	none	Bil-CRP: **0.0002**<br>CRP-Plt: **0.0005**<br>Na-Cre: **0.0019**<br>Na-PF: **0.0043**<br>MAP-Na: **0.0066**<br>MAP-CRP: **0.0113**<br>Na-CRP: **0.0136**	none

S4 Supplementary results: Random control selection

S4.1 Setting

In our original analysis in the main text, we have derived the set of controls (i.e., the survivor group) based on an explicit procedure including a propensity score matching (PSM) step (see Figures 3 and 4 in the main text, along with the corresponding descriptions). To show the impact of control selection on our results, we here perform an additional analysis based on repeated random selection of the controls as follows. For each of our 123 finally selected cases (see Figure 4 in the main text), we randomly draw 100 times a control from the corresponding risk set obtained after the length-of-stay matching (compare Figure 3 in the main text) and in particular omit the PSM step. Hence, we obtain 100 control groups (survivor groups) consisting of 123 samples each, both for admission and event stage. For each set of controls, we re-perform our analyses for comparisons (C1), (C2) and (C3), see Figure 2 in the main text. Note that as the 123 cases are fixed, the non-survivors' admission and the non-survivors' event networks are fixed, and not randomized. Thus, the results for comparison (C4) from the main text are not affected by the random control selection.

In what follows, we briefly describe the set of the 100 random control groups and compare it to the PSM-based control group from the main text. As stated before, for each of the 123 cases, 100 random samples are drawn from the corresponding risk set, where each case has a risk set of a different size. In our specific setting, the risk set sizes range from 3 to 762, with a mean of 423 (standard deviation: 215) and a median of 440. As we draw 100 samples from each of the 123 risk sets, we can thus expect a reasonable degree of variability in the random draws. An overview of the composition of the 100 random control groups in comparison to the original, PSM-based control group is given in Figure S13. In particular, it can be observed that the random control draws are reasonably different from the PSM-based control group. Meaningfully, overlaps virtually only occur for controls associated to a case with a small risk set size (right hand-side in Figure S13). For instance, the PSM-based control with ID 123 (PSMCtrl_ID123 in Figure S13) is associated to a case with a risk set size of 3 (the smallest in our setting) and is present in 36 out of the 100 random control groups (and thus in approximately one third of the random control groups, as expected).

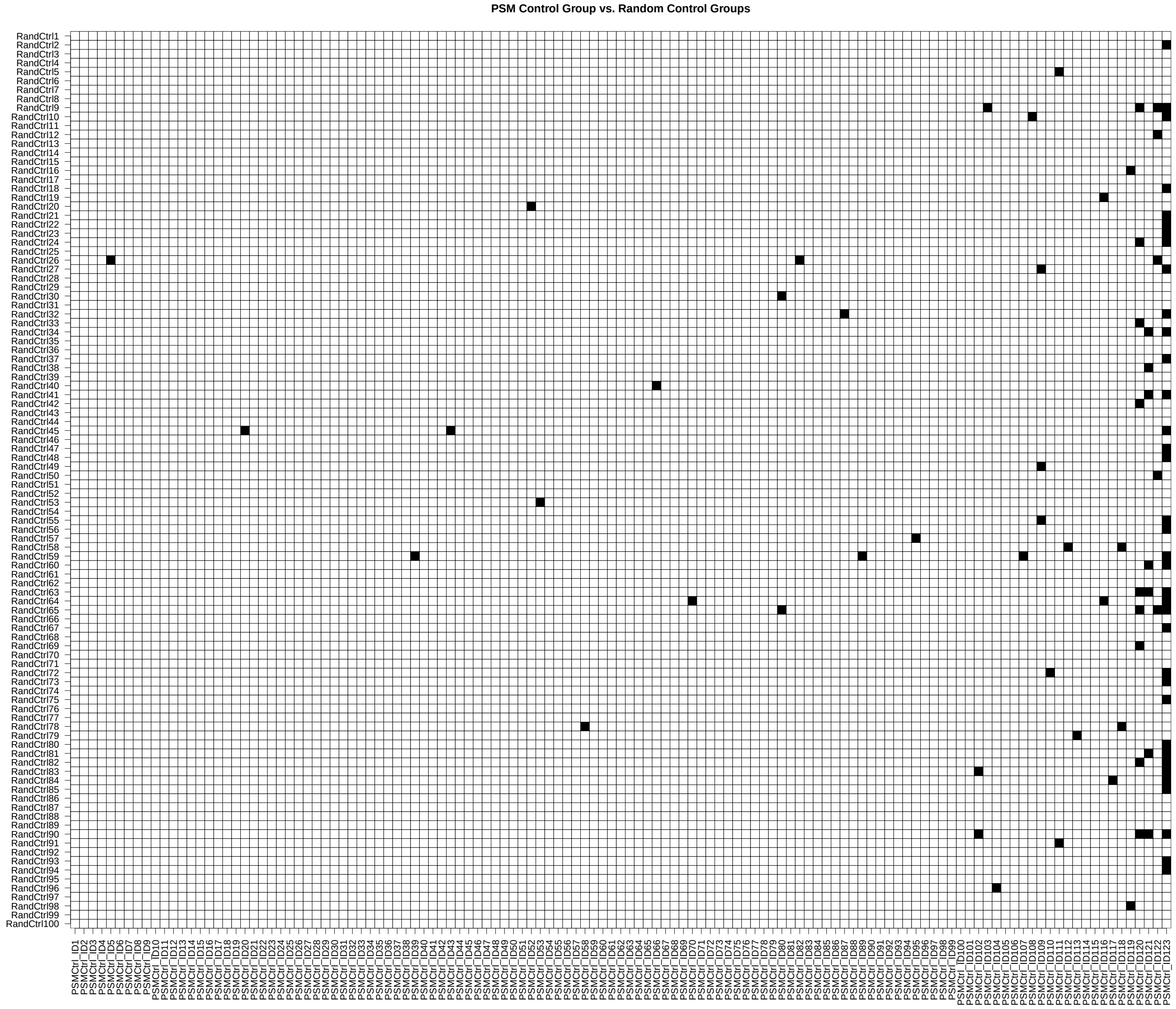

Figure S13: Overview of the composition of the 100 random control groups, each comprising 123 encounters, in comparison to the original, PSM-based control group also comprising 123 encounters: A grid point in the (123 × 100) matrix is filled in black if the corresponding encounter from the PSM control group (x-axis) is present in the corresponding random control group (y-axis) and left blank otherwise. Note that the 123 PSM-based control encounters (PSMCtrl_ID1 to PSMCtrl_ID123 on the x-axis) are arranged in descending order with respect to the risk set size of the corresponding case.

S4.2 Results for Spearman correlations with Bonferroni adjustment

We here summarize the differential network testing results for the comparisons (C1), (C2) and (C3) for networks estimated using Spearman correlations together with Bonferroni adjustment, where we compare the corresponding p-values obtained using the 100 random control groups to that obtained using the PSM control group (Figures S14 to S16).

Generally, there appears to be quite some heterogeneity in the findings, in that some of the results for the PSM control group are confirmed, and some are not.

Regarding comparison (C1), i.e. non-survivors at admission vs. survivors at admission, the results obtained for the PSM control group basically continue to hold for the majority of the random control groups. That is, also for random control groups, there essentially does not appear to be a significant difference between the non-survivor and the survivor group at admission. However, the p-values associated to the PSM control group are typically (very) high throughout, while for the random control groups also lower p-values (even ≤ 0.05) can be observed. Hence, the PSM in any case guarantees that the admission networks for non-survivors and survivors are similar, i.e. the starting point of our original analyses.

For comparison (C2), i.e. non-survivors at event vs. survivors at event, partly differing results for the PSM control group and the random control groups can be observed, depending on the considered network difference characteristics. While for the PSM control group all p-values relating to overall network difference characteristics are low (and often ≤ 0.05), this does not fully hold for the random control groups. Here, the corresponding median p-values are > 0.05 except for that of the Jaccard distance. For the overall network difference characteristics, the p-values related to the PSM control group are lower than the median p-values for the random control groups. Concerning differences with respect to node degree, for CRP, which shows a significantly different degree for the PSM control group, the median of the p-values for the random control groups is slightly above, but close to the significance level of 5%. Interestingly, and in contrast to the results for the PSM control group, sodium shows a significantly different degree for the random control groups, with a median p-value ≤ 0.05. Regarding differences in terms of edge strength, the results appear to be quite robust, in that, as for the PSM control group, the edges Bil-CRP, Bil-Na and Na-Plt are associated with median p-values ≤ 0.05 for the random control groups.

Finally, with respect to comparison (C3), i.e. survivors at admission vs. survivors at event, the median p-values for the random control groups indicate non-significant differences for all overall network difference characteristics and the node degree, in contrast to the results obtained for the PSM control group. However, the prominent difference with respect to the edge Bil-CRP is confirmed, in that the corresponding median p-value for the random control groups is ≤ 0.05. A possible explanation for the non-significant differences of the random control groups with respect to overall network difference characteristics in comparison (C3), in contrast to the PSM control group, may be as follows. Regardless of the control selection method, the possible controls do not appear to differ that much with respect to the nine network parameters at admission stage, which is witnessed by the results for comparison (C1) as discussed before. However, the PSM-based approach guarantees to select controls that are not only similar to the cases in terms of the network parameters, but additionally regarding overall clinical condition and comorbidities at ICU admission. This is not addressed when using randomly selected controls. With that said, the control patients selected by the PSM-based approach can be expected to be generally in a more critical clinical condition (as the cases are) than those chosen randomly. Thus, it appears to be more likely to observe a dynamic towards stabilization for the PSM control group (main text) than for the random control groups, for which the organ system interactions overall appear to remain basically unchanged.

Overall, the results thus confirm the utility and validity of the PSM-based control selection procedure from the main text to perform comparisons that are designed as fairly and meaningfully as possible.

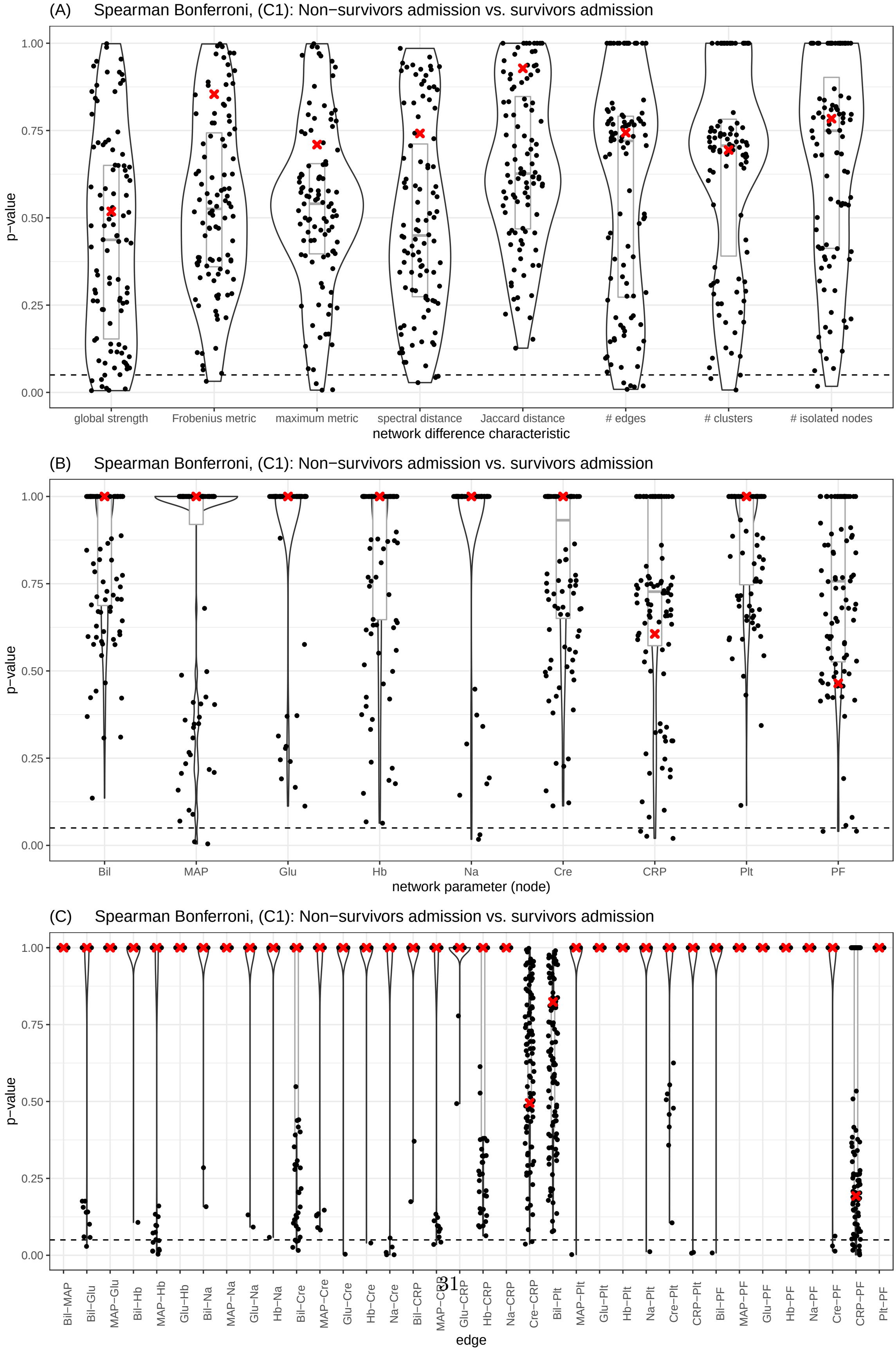

Figure S14: P-values obtained when testing for differences with respect to (A) overall network difference characteristics, (B) node-specific degree, (C) edge-specific edge strength based on networks estimated using Spearman correlations together with Bonferroni adjustment for comparison (C1), i.e. non-survivors at admission vs. survivors at admission. Black dots: p-values for the comparisons based on the 100 random control groups; gray: corresponding boxplots; red cross: p-value when using PSM control group; dashed line: 5% significance level.

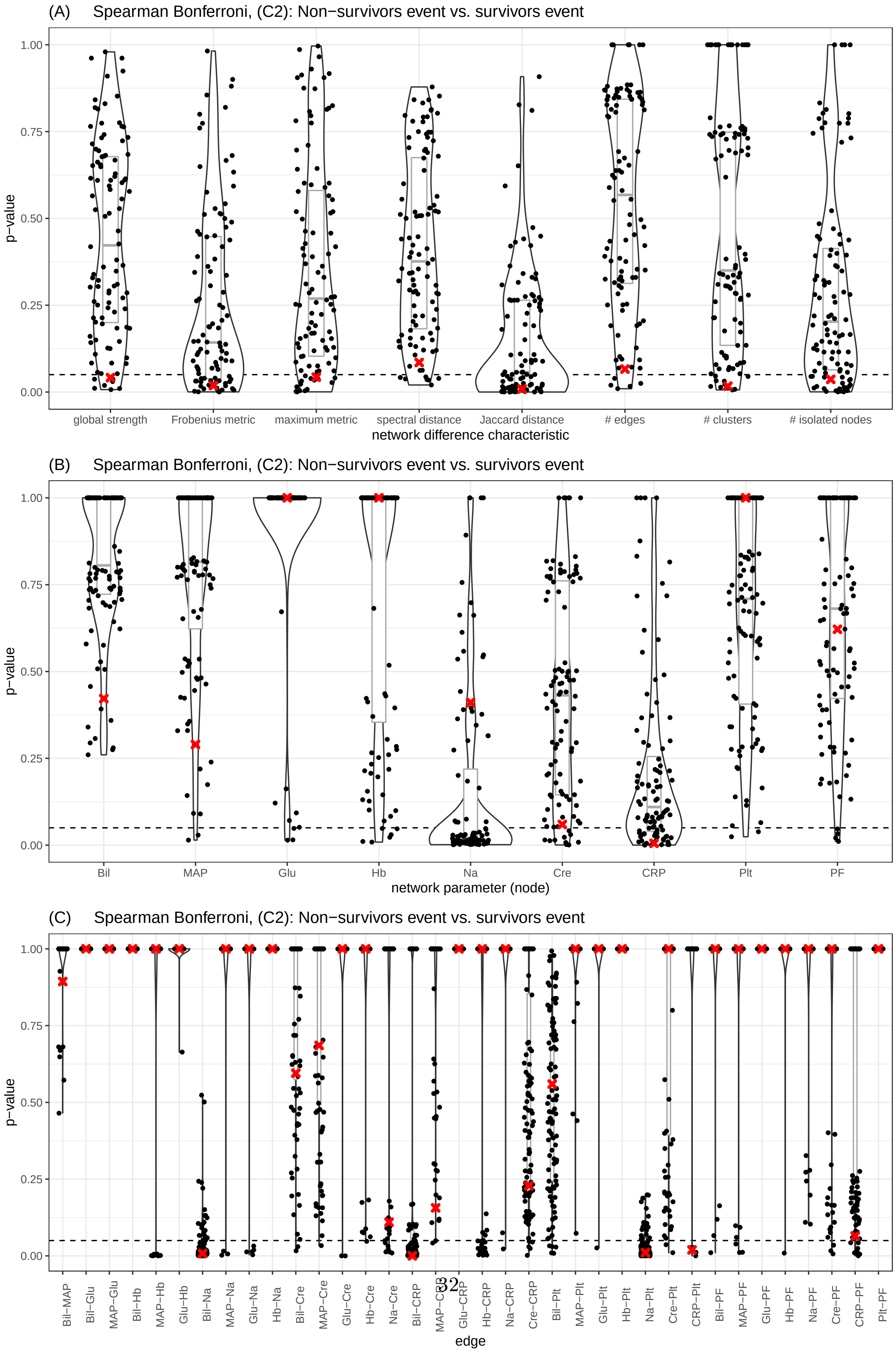

Figure S15: P-values obtained when testing for differences with respect to (A) overall network difference characteristics, (B) node-specific degree, (C) edge-specific edge strength based on networks estimated using Spearman correlations together with Bonferroni adjustment for comparison (C2), i.e. non-survivors at event vs. survivors at event. Black dots: p-values for the comparisons based on the 100 random control groups; gray: corresponding boxplots; red cross: p-value when using PSM control group; dashed line: 5% significance level.

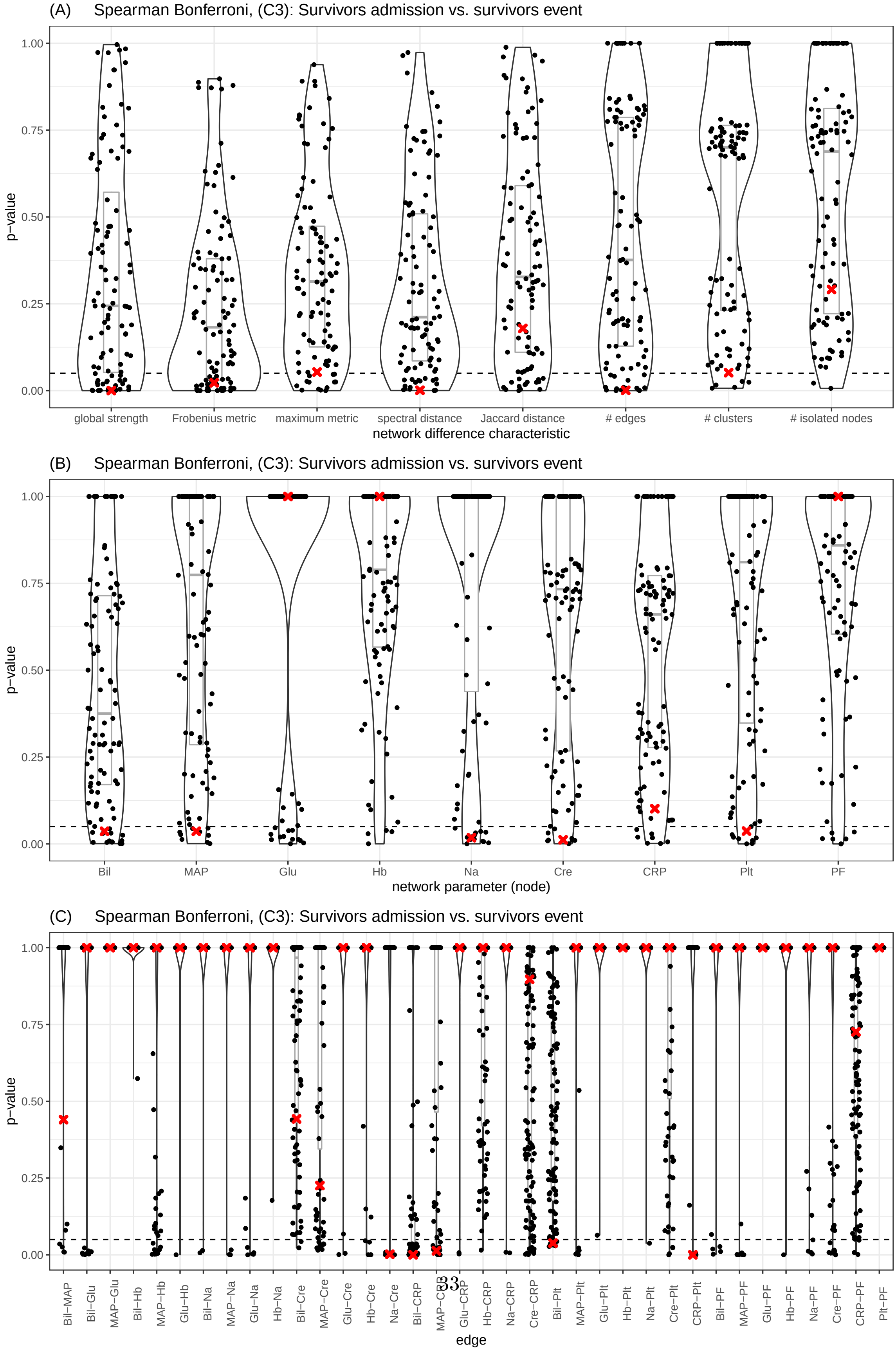

Figure S16: P-values obtained when testing for differences with respect to (A) overall network difference characteristics, (B) node-specific degree, (C) edge-specific edge strength based on networks estimated using Spearman correlations together with Bonferroni adjustment for comparison (C3), i.e. survivors at admission vs. survivors at event. Black dots: p-values for the comparisons based on the 100 random control groups; gray: corresponding boxplots; red cross: p-value when using PSM control group; dashed line: 5% significance level.

S4.3 Results for Spearman correlations with BH adjustment

For the sake of completeness, we here show the results of our additional analyses when using networks based on Spearman correlations with BH adjustment (Figures S17 to S19).
Basically, the results described before, obtained when using networks based on Spearman correlations with Bonferroni adjustment, continue to hold, with few exceptions only.

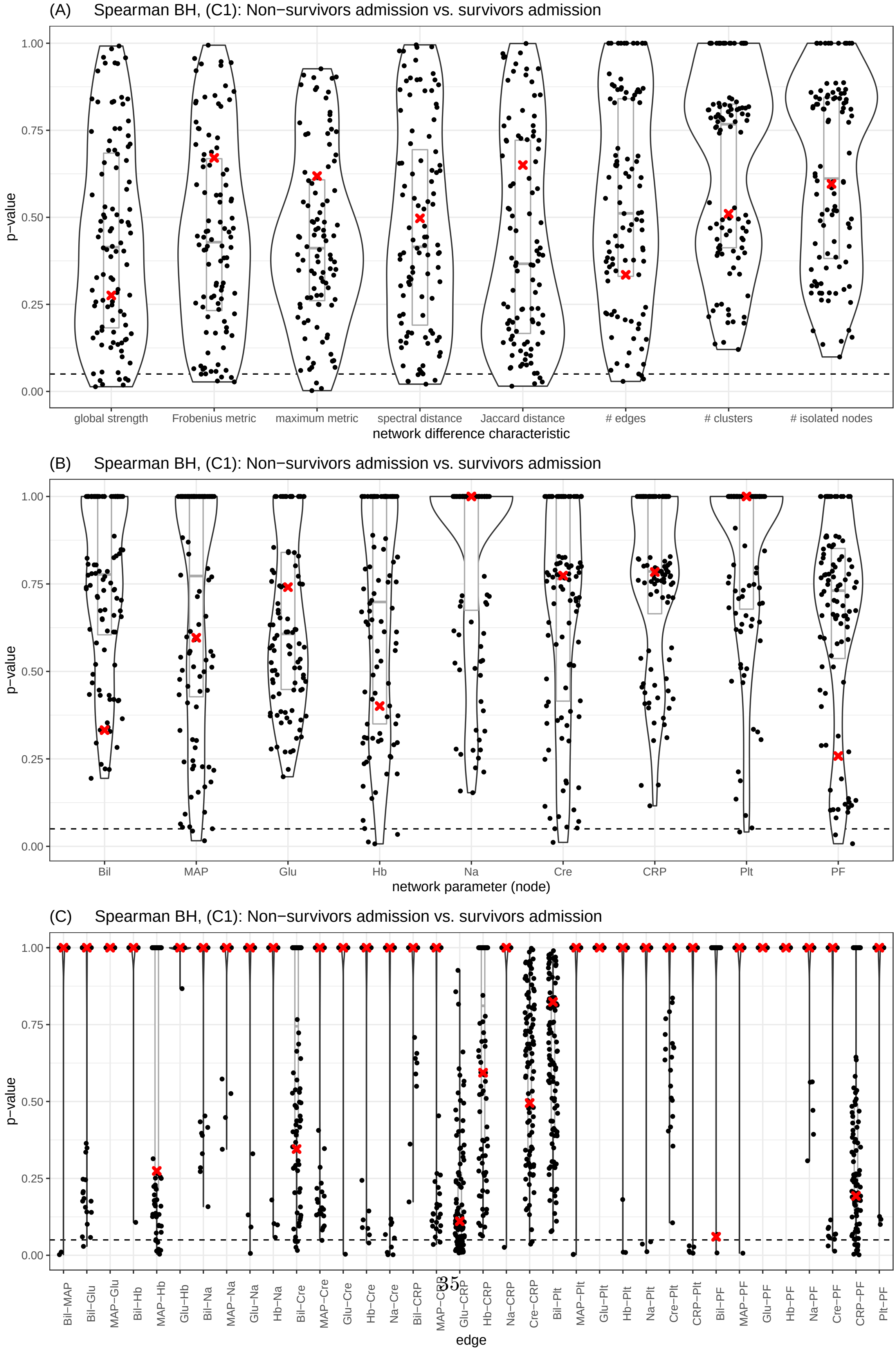

Figure S17: P-values obtained when testing for differences with respect to (A) overall network difference characteristics, (B) node-specific degree, (C) edge-specific edge strength based on networks estimated using Spearman correlations together with BH adjustment for comparison (C1), i.e. non-survivors at admission vs. survivors at admission. Black dots: p-values for the comparisons based on the 100 random control groups; gray: corresponding boxplots; red cross: p-value when using PSM control group; dashed line: 5% significance level.

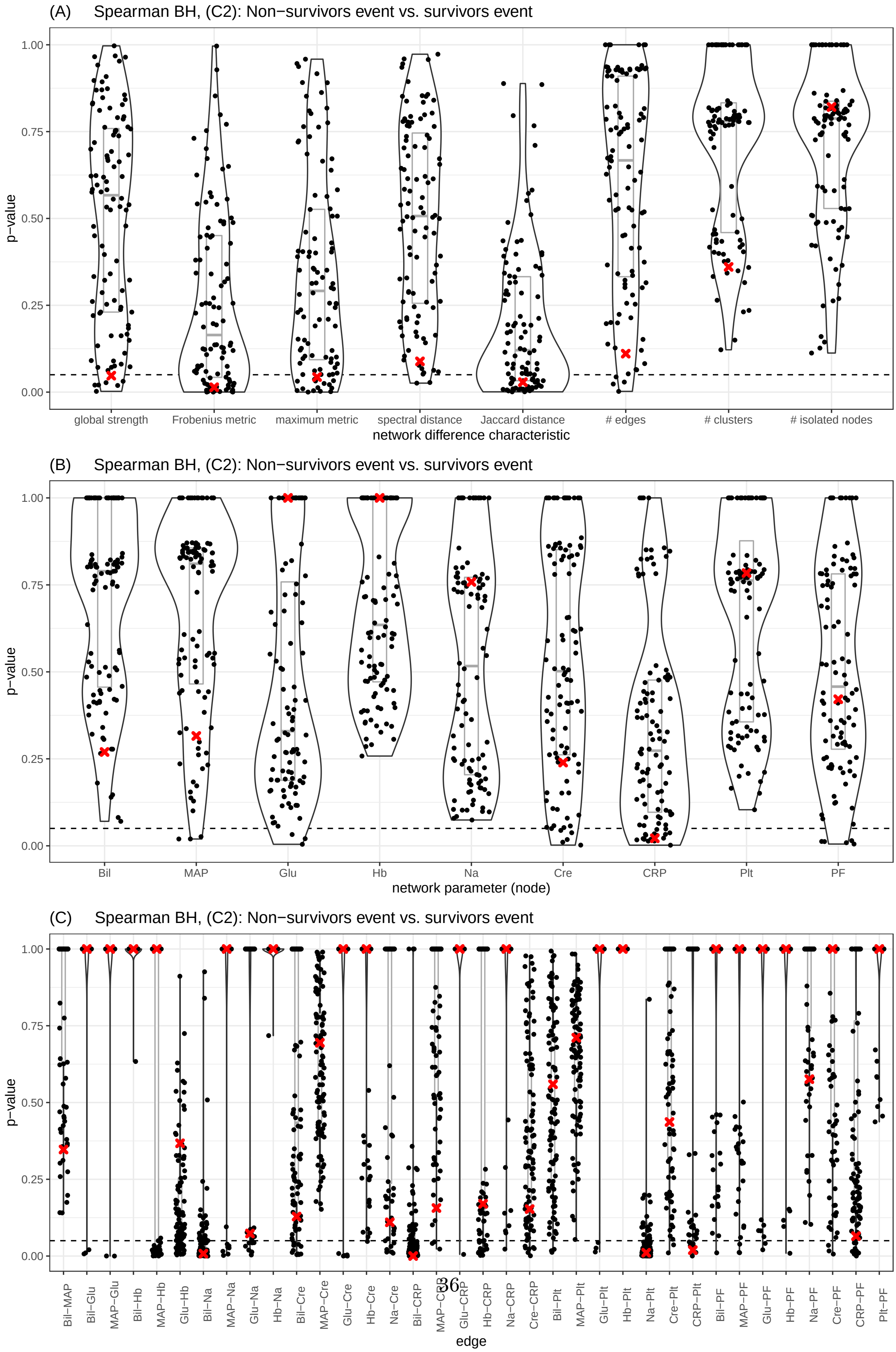

Figure S18: P-values obtained when testing for differences with respect to (A) overall network difference characteristics, (B) node-specific degree, (C) edge-specific edge strength based on networks estimated using Spearman correlations together with BH adjustment for comparison (C2), i.e. non-survivors at event vs. survivors at event. Black dots: p-values for the comparisons based on the 100 random control groups; gray: corresponding boxplots; red cross: p-value when using PSM control group; dashed line: 5% significance level.

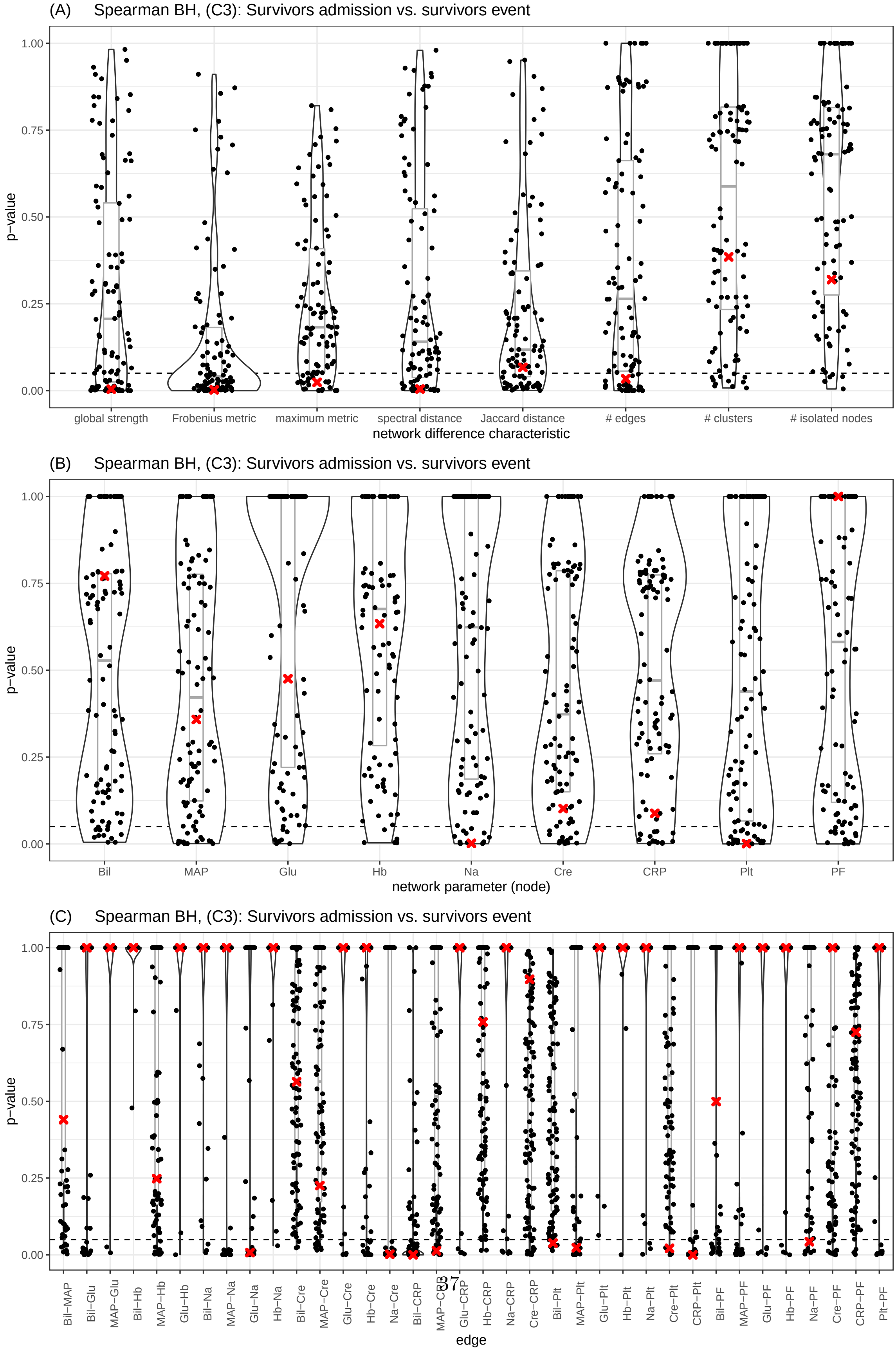

Figure S19: P-values obtained when testing for differences with respect to (A) overall network difference characteristics, (B) node-specific degree, (C) edge-specific edge strength based on networks estimated using Spearman correlations together with BH adjustment for comparison (C3), i.e. survivors at admission vs. survivors at event. Black dots: p-values for the comparisons based on the 100 random control groups; gray: corresponding boxplots; red cross: p-value when using PSM control group; dashed line: 5% significance level.

References

[1] J. T. Daugirdas and A. A. Bernardo. Hemodialysis effect on platelet count and function and hemodialysis-associated thrombocytopenia. *Kidney International*, 82:147–157, 2012.

[2] J. P. Kooman, F. Van Der Sande, K. Leunissen, and F. Locatelli. Sodium balance in hemodialysis therapy. *Seminars in Dialysis*, 16:351–355, 2003.
